# First-Principles Investigation of Phosphorus-Doped Graphitic Carbon Nitride as Anchoring Material for the Lithium-Sulfur Battery

**DOI:** 10.3390/molecules29122746

**Published:** 2024-06-09

**Authors:** Yuehui Chen, Fengxia Liu, Shuang Wei, Yingkai Xia, Xiaodong Li, Shengnan Liu, Xu Zhang, Shuwei Tang, Ding Shen, Wei Dong, Shaobin Yang

**Affiliations:** 1 School of Mining, Liaoning Technical University, Fuxin 123000, China; chenyuehui@lntu.edu.cn (Y.C.); liufengxia1981@126.com (F.L.); weishuangcoyi@163.com (S.W.); xiayingkai200719@126.com (Y.X.); 2 College of Science, Liaoning Technical University, Fuxin 123000, China; lsn0525@foxmail.com; 3 College of Material Science and Engineering, Liaoning Technical University, Fuxin 123000, China; 18955016823@163.com (X.L.); zhangxu@lntu.edu.cn (X.Z.); tangsw911@nenu.edu.cn (S.T.); shending@lntu.edu.cn (D.S.); lgddongwei@163.com (W.D.)

**Keywords:** first-principles, graphitic carbon nitride, polysulfide, lithium-sulfur battery

## Abstract

The utilization of lithium–sulfur battery is hindered by various challenges, including the “shuttle effect”, limited sulfur utilization, and the sluggish conversion kinetics of lithium polysulfides (LiPSs). In the present work, a theoretical design for the viability of graphitic carbon nitride (g-C_3_N_4_) and phosphorus-doping graphitic carbon nitride substrates (P-g-C_3_N_4_) as promising host materials in a Li-S battery was conducted utilizing first-principles calculations. The PDOS shows that when the P atom is introduced, the 2p of the N atom is affected by the 2p orbital of the P atom, which increases the energy band of phosphorus-doping substrates. The energy bands of P_C_ and P_i_ are 0.12 eV and 0.20 eV, respectively. When the lithium polysulfides are adsorbed on four substrates, the overall adsorption energy of P_C_ is 48–77% higher than that of graphitic carbon nitride, in which the charge transfer of long-chain lithium polysulfides increase by more than 1.5-fold. It is found that there are powerful Li-N bonds between lithium polysulfides and P-g-C_3_N_4_ substrates. Compared with the graphitic carbon nitride monolayer, the anchoring effect of the LiPSs@P-g-C_3_N_4_ substrate is enhanced, which is beneficial for inhibiting the shuttle of high-order lithium polysulfides. Furthermore, the catalytic performance of the P-g-C_3_N_4_ substrate is assessed in terms of the S_8_ reduction pathway and the decomposition of Li_2_S; the decomposition energy barrier of the P-g-C_3_N_4_ substrate decrease by 10% to 18%. The calculated results show that P-g-C_3_N_4_ can promote the reduction of S_8_ molecules and Li-S bond cleavage within Li_2_S, thus improving the utilization of sulfur-active substances and the ability of rapid reaction kinetics. Therefore, the P-g-C_3_N_4_ substrates are a promising high-performance lithium-sulfur battery anchoring material.

## 1. Introduction

The burgeoning demand for specific energy conversion and storage devices in contemporary society has spurred extensive exploration and research beyond the conventional lithium-ion battery [1,2,3]. The lithium-sulfur (Li-S) battery has emerged as a promising candidate for commercial lithium-ion batteries on account of its gravimetric energy density and theoretical specific capacity. The benefit of the Li-S battery lies in its low equivalent weight, cost-effectiveness, natural abundance, and minimal environmental impact. The charge/discharge processes of the Li-S battery proceed via a series of polysulfide intermediates (the LiPSs, Li_2_S_n_ (*n* = 1, 2, 4, 6, and 8)), accompanied by the gradual reduction of the S_8_ molecule. However, the high-order intermediates (Li_2_S_4_, Li_2_S_6_, and Li_2_S_8_) exhibit a tendency to dissolve in organic solvents, leading to the undesirable “shuttle effect”. As a consequence, the robust self-discharge, significant capacity loss, and depletion of active sulfur have coexisted in the Li-S battery [4,5].

In recent years, considerable endeavors have been directed towards mitigating the dissolution and diffusion of high-order polysulfides, involving physically confining the high-order LiPSs within the pores or layers of high-surface-area carbon nanostructures, such as carbons featured with micro or mesopores [6,7,8,9,10,11]. Nevertheless, these physical adsorption strategies often fail to efficiently address the “shuttle effect” because of the weak affinity of nonpolar carbon materials for high-order LiPSs. Recently, chemical adsorption has emerged as a viable strategy for LiPSs retention. The polar nanomaterials, including metal oxides [12,13,14], metal nitrides [15], transition metal sulfides [16,17], and metal-organic frameworks [18], have shown promising prospects in this regard. These materials featured with polar surfaces provide chemical anchoring sites for the LiPSs. Nevertheless, the utilization of metallic materials in Li-S batteries tends to reduce the energy density and increase the proportion of inactive materials in the composition of host materials. Very recently, considerable attention has been directed toward identifying compelling host materials for the confinement of S and the anchoring of the LiPSs, with particularly effort focused on the two-dimensional (2D) materials renowned for their high surface areas, active adsorption sites, and porous configurations [19]. Graphitic carbon nitride (g-C_3_N_4_) [20,21,22,23,24], as a lightweight, polar, highly ordered polymeric material, showcases an excellent prospect for enhancing the capacity and cycling performance of the Li-S battery. Within the crystal structure of the g-C_3_N_4_ monolayer, the sp^2^ hybridized nitrogen atoms with lone pair electrons are separated by carbon atoms, showcasing some delocalization. Meanwhile, one sp3 hybridized nitrogen atom forms a covalent bond with three adjacent carbon atoms [25,26,27,28]. However, the intrinsically poor electrical conductivity of g-C_3_N_4_ limits the Li-S battery’s performance. Chemical doping has proven to be an effective strategy for altering semiconductors’ electronic structures and surface characteristics. For instance, Zhang et al. demonstrated a significant enhancement of electrical conductivity through the phosphorus doping polymeric g-C_3_N_4_ [29,30]. Similarly, Ma et al. significantly improved the electrochemical performance of phosphorus-doping graphitic carbon nitride [31]. These findings indicate that the phosphorus-doping graphitic carbon nitride (P-g-C_3_N_4_) is a promising anchor host material for Li-S batteries. However, the doping configuration for P-g-C_3_N_4_ and the anchoring mechanism have not been identified clearly in the theoretical and experimental work on the Li-S battery. Therefore, a comprehensive investigation of the P-g-C_3_N_4_ configuration of the anchoring mechanism of S_8_ and LiPSs interacting with P-g-C_3_N_4_ substrates is imperative for addressing this issue.

In the present work, a comprehensive exploration of the interactions between the LiPSs and substrates (the g-C_3_N_4_ and P-g-C_3_N_4_) is undertaken through first-principles calculations. The diverse doping configurations of P-g-C_3_N_4_, involving the substitutional and interstitial doping sites, are considered. S_8_ and LiPSs species exhibit favorable adsorption energy for P-g-C_3_N_4_, underscoring the suitability of phosphorus-doped graphitic carbon nitride as a moderate host material for suppressing the shuttle effect of the high-order LiPS species. Therefore, the P-g-C_3_N_4_ substrates emerge as a promising host material for high-performance Li-S batteries.

## 2. Results and Discussion

### 2.1. Structures for S_8_, LiPSs Species, g-C_3_N_4_ and P-Doping g-C_3_N_4_

**Figure 1:**
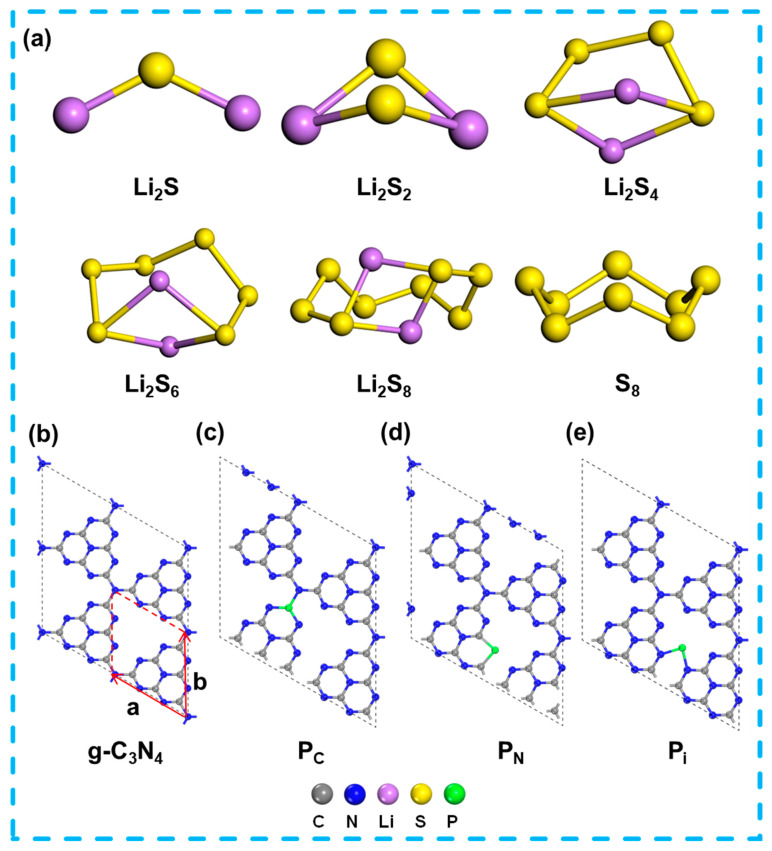
(**a**) Optimized structures of the isolated S_8_, LiPSs (Li_2_S_n_, n = 1, 2, 4, 6 and 8) species. (**b**–**e**) Optimized structures of the g-C_3_N_4_ and P-doping g-C_3_N_4_ monolayer.

The optimized structures for the LiPSs are shown in Figure 1 and Figure S1 of the Supporting Information (SI). All calculations have been implemented for the heptazine-based graphitic carbon nitride (g-C_3_N_4_) with the 2 × 2 × 1 supercell. Figure 1a and Figure S1a present the optimized structures of S_8_, LiPSs (Li_2_S_n_, *n* = 1, 2, 4, 6, and 8) clusters with point groups and lattice parameters. The S_8_ molecule adopts a puckering ring configuration with a *D*_4*d*_ point group, with an S–S bond length and an angle of 2.059 Å and 109.2°, respectively. For the LiPSs, the Li_2_S and Li_2_S_2_ display the *C*_2*v*_ point group, while the absence of mirror symmetry results in *C*_2_ symmetry for the Li_2_S_4_, Li_2_S_6_, and Li_2_S_8_ molecules. Within the LiPSs clusters, the shortest Li–S bond length increases as the rising cluster size: 2.108 Å for Li_2_S, 2.247 Å for Li_2_S_2_, and 2.388 (2.425 and 2.414) Å for Li_2_S_4_ (Li_2_S_6_ and Li_2_S_8_), while a reduced tendency is observed for the Li–S–Li bond angle, namely, 126.1° for Li_2_S, 96.5° for Li_2_S_2_, and 72.2° (66.1° and 67.7°) for Li_2_S_4_ (Li_2_S_6_ and Li_2_S_8_). The optimized structures of S_8_ and the LiPSs (Li_2_S_n_, *n* = 1, 2, 4, 6, and 8) molecules are in line with previous calculations [32,33,34,35].

The graphite-like g-C_3_N_4_ monolayer, characterized by an N-linked heptazine unit, prefers to crystallize in the hexagonal configuration (Figure 1b), in line with the findings of Thomas et al. [28,36,37,38,39]. In the structure of the g-C_3_N_4_ monolayer, the bridge and inner N atoms are 3-fold coordinated by three C atoms, whereas the edge N atoms are only 2-fold coordinated by two C atoms. A limitation of DFT-based means is their insufficient resolution on weak vdW interactions, which are essential for compounds resembling graphite [40].

To obtain an accurate configuration of the g-C_3_N_4_ monolayer, a theoretical comparison of the lattice parameters of the g-C_3_N_4_ monolayer is conducted, as depicted in Table 1. The experimental data is also presented for comparison. The results show that the lattice parameter (a = 7.072 Å) calculated by the LDA-CAPZ method [41,42] without vdW correction and the lattice parameter (a = 7.138 Å) calculated by PBE-TS dispersion correction [34] are smaller than the results of the PBE-Grimme (DFT-D2) dispersion; the latter parameters (a = 7.144 Å) are closer to the experimental value (a = 7.300 Å) [32], and PBE–Grimme’s calculations are in better agreement with the theoretical results of the g-C_3_N_4_ monolayer in ref. [36,38].

### 2.2. Formation Energies of P-Doping g-C_3_N_4_

The 2×2×1 supercell with 24 C and 32 N atoms is employed for P-doping g-C_3_N_4_; p can replace the C/N atoms or form an interstitial atom. To determine the most stable configuration of the P-doping g-C_3_N_4_ system, nine distinct P-doping configurations are evaluated in terms of formation energy. The designations of N1-N4 and C1-C4 denote four N and four C sites, respectively, while Pi represents the interstitial doping site, as illustrated in Figure S1 of SI. The formation energy is calculated using Equations (1) and (2), which represent substitutional and interstitial doping, respectively [38,43,44]. The lower defect formation energy *E**_f_*** indicates greater defect concentration. Generally, the defect configuration with low formation energy exhibits great tendency information in the structure of the g-C_3_N_4_ monolayer. The following equations, (1) and (2), are adopted to calculate the formation energy of substitutional and interstitial dopants:

(1) $$E_{f}=E_{tot}(sub-dopant)-E_{tot}(pure)-\mu_{A}-\mu_{B}$$

(2) $$E_{f}=E_{tot}(\mathrm{int}-dopant)-E_{tot}(pure)-\mu_{A}$$

where the $E_{tot}(sub-dopant)$ and $E_{tot}(\mathrm{int}-dopant)$ represents the total energies for the substitutional and interstitial dopants, respectively. The $E_{tot}(pure)$ denotes the total energies of g-C_3_N_4_. The $\mu_{A}$ and $\mu_{B}$ denote the atomic potentials of the P and C/N atoms, respectively. The $\mu_{C}$ and $\mu_{N}$ are estimated from the energy of the graphite and a nitrogen molecule ($\mu_{N}=1/2\mu_{N_{2}}$), respectively. The $\mu_{P}$ is determined from white phosphorus [43,44]. In general, thermodynamic factors such as vibration and enthalpy should be considered for the free energy calculations; however, due to the limitation of the workstation, the electronic energies are adopted to evaluate the formation energies without considering the influence of temperature [45]. These results are consistent with the reports in refs. [38].

The g-C_3_N_4_ structure comprises four N sites, four C sites, and one interstitial site, as shown in Figure S1 of SI. Specifically, both substitution and interstitial doping are accounted for in the mono-doping strategy. Several possible P-doping g-C_3_N_4_ configurations, including three inequivalent N sites (N1, N3, N4), two inequivalent C sites (C1, C2), and one interstitial site (P_i_), are obtained, and the corresponding formation energies are shown in Table 2. Lower defect formation energy indicates that impurity ions prefer to be incorporated into the substrate [38,46]. Among the doping sites, the defect formation energies of g-C_3_N_4_ with the interstitial site (P_i_) are the lowest, implying that the interstitial site is in the optimal position. Such a phenomenon can be attributed to the isoelectronic nature of P with N, whereas C introduces only one electron into the g-C_3_N_4_ monolayer, leading to destabilization [34].

Some previous experimental findings suggest that P atoms primarily substitute C1 atoms within the g-C_3_N_4_ monolayer before replacing other positions [30], which indicates that a portion of P atoms should be adsorbed within the ample space of the planar structure. Upon P_i_ doping, one lone pair electron localizes around the P atom, while another electron delocalizes around the N–P–N chain. Two sp^2^ hybrid orbitals of P form bonds with two sp^2^ hybrid orbitals of adjacent N atoms. The configuration of the C–N=C chain results from the interplay between the energy gained from extending the π electron and the repulsive force exerted by the lone pair electrons on the N-side atoms in the heptazine unit [39,40]. The formation energy of P-g-C_3_N_4_ is consistent with previous theoretical studies [37,38]. Due to the slight difference in formation energies for the replacement P atom on the N and C sites, three distinct doping configurations are also considered, as depicted in Table 2. The (2 × 2 × 1) and (4 × 4 × 1) defect formation energies of the supercells are calculated in Table 2, and the results show that the substitution formation energies at the C/N position are the same as those at the interstitial position. Considering that the focus of our present work is to study the interaction between g-C_3_N_4_ and polysulfides from the beginning of the monolayer and to study the possibility of doped P elements as a base-anchoring material, and considering factors such as the number of atoms, the amount of work, and the ability to calculate, we choose (2 × 2 × 1) supercells for the subsequent calculations. In Table 2, the highest formation energy is found at the N4 position, and the comparison reveals that Ma et al. use a bilayer AB stacked structure different from the two-dimensional monolayer material in this paper, and the computational functions, parameter settings, and vacuum layers are the difference. The substitution and interstitial position pattern after doping with P elements is consistent with refs. [38,39]. The results show the possibilities of positions including the interstitial site (P_i_) (*E_f_* = 0.81 eV), and substituting sites which involved the C1 position (P_C_) (*E_f_* =1.06 eV) and N3 position (P_N_) (*E_f_* =1.34 eV). Figure 1b–e presents the different configurations of the P-doping g-C_3_N_4_ monolayer.

Figures S2 and S3 of SI present the electronic band structures and partial density of states (PDOS) of pristine g-C_3_N_4_ and P-g-C_3_N_4_ (P_C_, P_N_, and P_i_), respectively, and the corresponding band gaps (*E*_g_) are listed in Table 3. The g-C_3_N_4_ is a direct band gap semiconductor with an *E*_g_ of 2.67 eV, comparable to the experimental value of 2.70 eV [46]. However, within the P-g-C_3_N_4_ (P_C_, P_N_, and P_i_), the electronic band structures vary significantly on account of the incorporation of the P atom. Compared with the g-C_3_N_4_ monolayer, the Fermi level of P-g-C_3_N_4_ (P_C_, P_N_, and P_i_) shifts downward toward the conduction band, resulting in reduced band gaps of 2.14 eV, 0.12 eV, and 0.20 eV for the P_N_, P_C_, and P_i_ configurations, respectively. These results align well with the experimental results of Zhang et al. [29], indicating a noticeable improvement in the electrical conductivity of P-g-C_3_N_4_. Introducing P elements in place of C/N is beneficial for generating lone pair electrons, thereby increasing the carrier concentration and potentially reducing the mobility gap [47]. This shift could facilitate movement towards the conjugated ring, affecting the electronic properties through quantum vibrational effects [48,49], which influences the electrical conductivity. These outcomes are consistent with prior research [50,51].

The DOS and PDOS in Figure S3 of SI reveal that the reduced band gaps are primarily attributed to the P-doping, particularly for the replacement of the C position, leading to a small band gap of 0.12 eV. Further PDOS analysis shows that the P 2s and 2p orbitals provide a dominant contribution near the Fermi level, accompanied by the 2p orbitals of N. A similar trend is observed for the situation of P_i_, where P 2p orbitals play a significant role in reducing the band gap. Therefore, replacing the N position with P results in a decreased band gap. Meanwhile, the small gap (*E*_g_ = 0.12 eV) of the P_C_ substrate is beneficial for enhancing the electrical conductivities of the LiPSs@P_C_ system.

### 2.3. Anchoring Structure and Adsorption Energy

**Figure 2:**
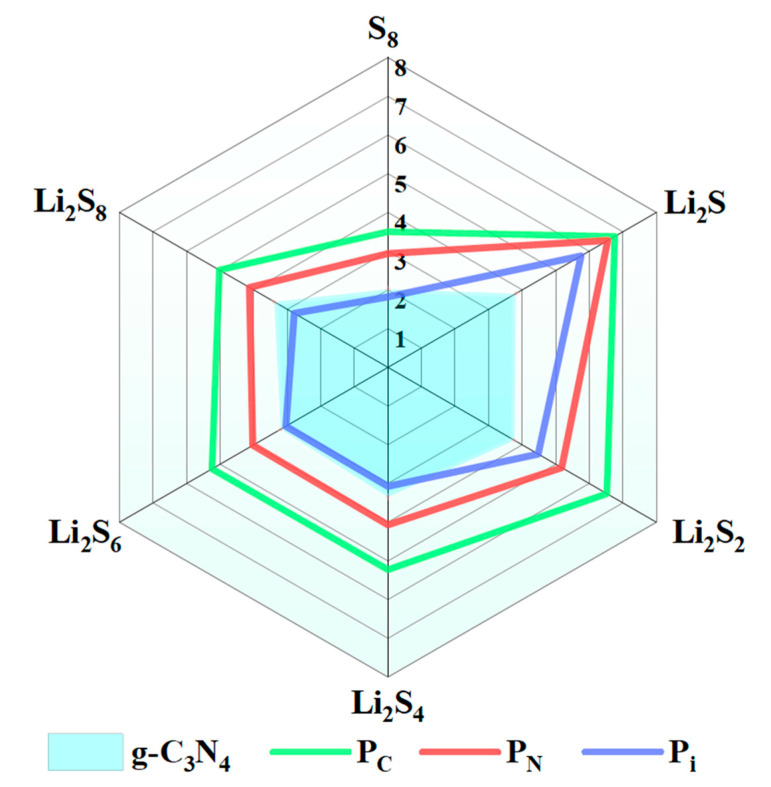
Adsorption energies (*E_ad_*) for the most stable S_8_ and Li_2_S_n_ molecules on the surface of g-C_3_N_4_, P_C_, P_N_, and P_i_ with the PBE functional.

To assess the anchor effect of S_8_ and the LiPSs on the g-C_3_N_4_ and P-doping g-C_3_N_4_ substrates (P_C_, P_N_, P_i_), the adsorption energy [43] (*E_ad_*) between the Li_2_S_n_ species and the substrates is determined by the following equation.

(3) $$E_{ad}=E_{S_{8}/LiPSs}+E_{sub}-E_{S_{8}/LiPSs@sub}$$

where $E_{S_{8}/LiPSs}$, *E_sub_*, and $E_{S_{8}/LiPSs@sub}$ denote the total energies of S_8_ and the LiPSs, the substrates system (g-C_3_N_4_, P_C_, P_N_, P_i_), and S_8_ and the LiPSs on the substrates, respectively. Obviously, a significant positive value suggests a high adsorption capability. The *E_ad_* values for the most stable adsorption configurations of S_8_ or Li_2_S_n_ and monolayer substrates (g-C_3_N_4_, P_C_, P_N_, and P_i_) [44] are discussed in detail in the following section. The radar chart (Figure 2) depicts the adsorption energies for the anchoring of S_8_ or Li_2_S*_n_* on the surface of the g-C_3_N_4_, P_C_, P_N_, and P_i_ substrates, and the corresponding structures are listed in Table S1 of SI. The *E_ad_* values for S_8_ anchoring on the g-C_3_N_4_, P_C_, P_N_, and P_i_ surfaces fall within the 1.82–3.50 eV range. As the lithiation process initiates, significant variations are discovered for the adsorption processes of the LiPSs on the surfaces of the g-C_3_N_4_, P_C_, P_N_, and P_i_ compounds. Specifically, for the lithiation stages from Li_2_S_8_ to Li_2_S_4_, P_i_ exhibits robust adsorption energies (2.79 to 3.07 eV). However, as the lithiation further occurs, substantially higher *E_ad_* values are observed for the P_i_ situation (4.48 to 5.75 eV) as compared to the counterparts of g-C_3_N_4_ (3.77 to 3.81 eV). Meanwhile, the *E_ad_* values of P_C_ and P_N_ are significantly higher than those of g-C_3_N_4_ (Table S2 of SI).

P_C_, P_N_, and P_i_ exhibit similar adsorption intensities, particularly for the P_C_ substrate, which emerges as the most favorable substrate for anchoring the LiPSs. The adsorption energy of the LiPSs@P_C_ increases gradually with the decrease in the proportion of the S element. A similar tendency is also discovered for the P_N_ and Pi situations. Therefore, compared with g-C_3_N_4_, the P_C_, P_N_, and P_i_ exhibit superior anchor interactions with the LiPSs, which are beneficial for reducing the shuttle effect during the charging and discharging processes. Compared with the carbon-based matrix, the polar pore structure of P-doping g-C_3_N_4_ is more favorable for the adsorption of S_8_ and the LiPSs [51,52,53,54].

Table S1 of SI presents the most favorable structures of S_8_ and the LiPSs anchoring on the g-C_3_N_4_, P_C_, P_N_, and P_i_ substrates. The corresponding adsorption energies and the average S-S, Li-S, and Li-N distance within the S_8_ and LiPSs adsorption on substrates are listed in Table 4 and Tables S2 and S3 of SI. The average Li-S bond length increases to about 0.2 Å due to the LiPSs adsorbed on the four substrates. However, the Li-N bond length decreases with the increasing adsorption energy, especially the Li-N bond length decreases to 1.9 Å in the short-chain LiPSs@P-g-C_3_N_4_. It can be seen that in the same concentration of elemental P-doping substrates, P, in addition to increasing the electrical conductivity of the substrate, is also more able to enhance the Li-N bond and the formation of chemical adsorption [42].

### 2.4. Charge Transfer and Electronic Properties

To obtain a comprehensive understanding of the interaction between the LiPSs species and substrates (g-C_3_N_4_, P_C_, P_N_, and P_i_), the charge density differences after adsorption are evaluated using the following equation:

(4) $$\Delta \rho =\rho_{{(S}_{8}/\mathrm{LiPSs}\text{@}\mathrm{sub})}-\rho_{\mathrm{sub}}-\rho_{S_{8}/\mathrm{LiPSs}}$$

where $\rho_{{(S}_{8}/\mathrm{LiPSs}\text{@}\mathrm{sub})}$, $\rho_{\mathrm{sub}}$, and $\rho_{S_{8}/\mathrm{LiPSs}}$ represent the electron density of S_8_ or the LiPSs anchoring on the substrates (g-C_3_N_4_, P_C_, P_N_, and P_i_), pristine substrates, and the S_8_ or LiPSs molecules, respectively.

**Figure 3:**
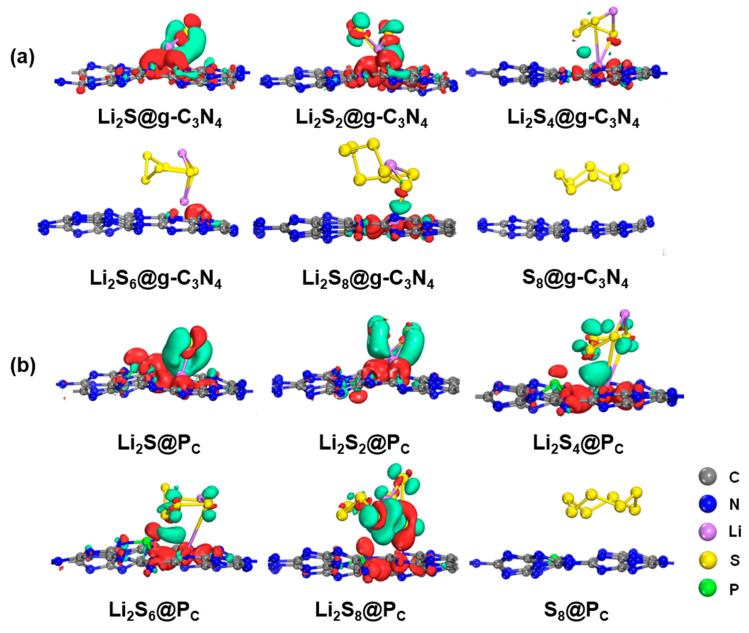
Charge transfer of S_8_ and LiPSs molecules adsorbed on (**a**) g-C_3_N_4_ and favorable (**b**) P_C_ substrates with the PBE functional. The green and red isosurfaces represent electron density loss and gain regions. The charge isosurfaces are 0.02 e/Å^3^ of S_8_ and Li_2_S_n_. (*n* = 1, 2,4, 6, and 8).

As presented in Figure 3 and Figure S4 of SI, minimal charge transfer occurs between the substrate and S_8_, indicating the absence of a chemical bond formation. However, upon the LiPSs anchoring on the substrates, a significant charge transfer occurs within and between the LiPSs. Specifically, there is an increase in the charge transfer between Li atoms and N, P, or C atoms. At the same time, a decrease is observed in the S atoms adjacent to Li atoms, leading to the formation of Li-N, C-S, or Li-P chemical bonds. This behavior is scrutinized across g-C_3_N_4_ and P-doping substrates (P_C_, P_N_, P_i_), as Figure S4 of SI depicts. A reinforcement of the Li-N bond and a weakening of the Li-S bond is discovered because of the strong electrostatic interaction between the positively charged Li atoms and negatively charged N atoms. Moreover, as the Li atom is introduced, the charge density between the LiPSs and the substrates increases inversely with the S content, particularly for the P_C_ substrate. This finding aligns well with the adsorption energy analysis. As shown in Figure 3, the distortion of the LiPSs species indicates that there is competition between the Li-N and Li-S chemical bonds, which is affected by charge transfer and changes in electronic structure, indicating a strong chemical adsorption between the LiPSs and the substrate [52,53,54].

**Figure 4:**
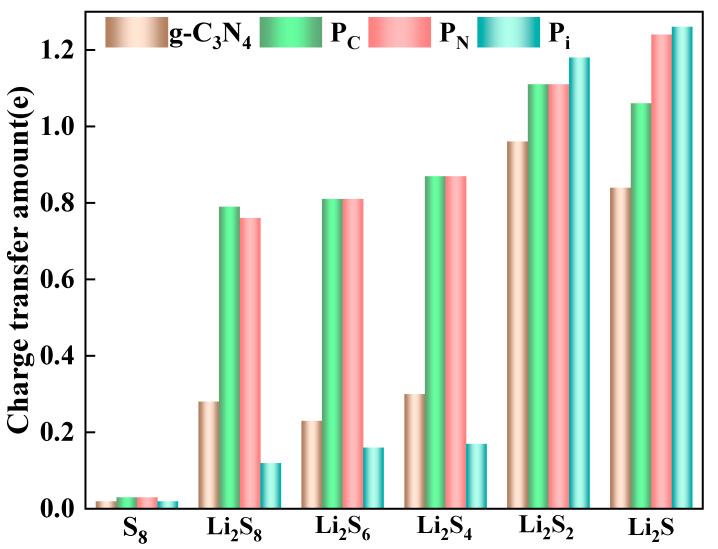
Charge transfer of S_8_ and Li_2_S_n_ (n = 1, 2, 4, 6, and 8) molecules adsorption on the surfaces of g-C_3_N_4_, P_C_, P_N_, and P_i_ substrates with the PBE functional.

To quantify the charge transfer between the g-C_3_N_4_, P_C_, P_N_, and P_i_ substrates and the S_8_ or LiPSs, the Mulliken charge analysis [55,56,57] is conducted, as depicted in Figure 4. The electron density transfers from the LiPSs to the substrates, which increases the positive charge of the LiPSs. However, due to the weak interaction between S_8_ and the four kinds of substrates, the charge transfer values for S_8_ molecules are comparatively small. Concurrently, enhancing the chemical interaction between the substrate and the LiPSs contributes to the elongation of the Li-S bond. For g-C_3_N_4_, the charge transfer for the adsorbed Li_2_S, Li_2_S_2_, Li_2_S_4_, Li_2_S_6_, and Li_2_S_8_ molecules are 0.84 e, 0.96 e, 0.30 e, 0.23 e, and 0.28 e, respectively, which correlates positively with the adsorption energy analysis as abovementioned. A similar trend is observed in P-doping situations, particularly for P_C_ substrates. The corresponding charge transfers are 1.06 e, 1.11 e, 0.87 e, 0.81 e, and 0.76 e for Li_2_S, Li_2_S_2_, Li_2_S_4_, Li_2_S_6_, and Li_2_S_8_, respectively. The Mulliken charge is known to be sensitive to the choice of basis set. To provide a more robust analysis, we also calculate the Hirshfield charge [56,58] (Table S4 of SI). The results corroborate the charge transfer analysis presented in Figure 3 and the Mulliken charge shown in Figure 4.

In general, adsorption strength directly correlates with the magnitude of charge transfer in host materials for Li-S batteries. Consequently, the anchoring capability of P-g-C_3_N_4_ substrates surpasses that of g-C_3_N_4_. Moreover, the LiPSs molecules gain electrons during the discharging process, leading to the transformation of long-chain intermediates of the LiPSs into short-chain counterparts and vice versa.

**Figure 5:**
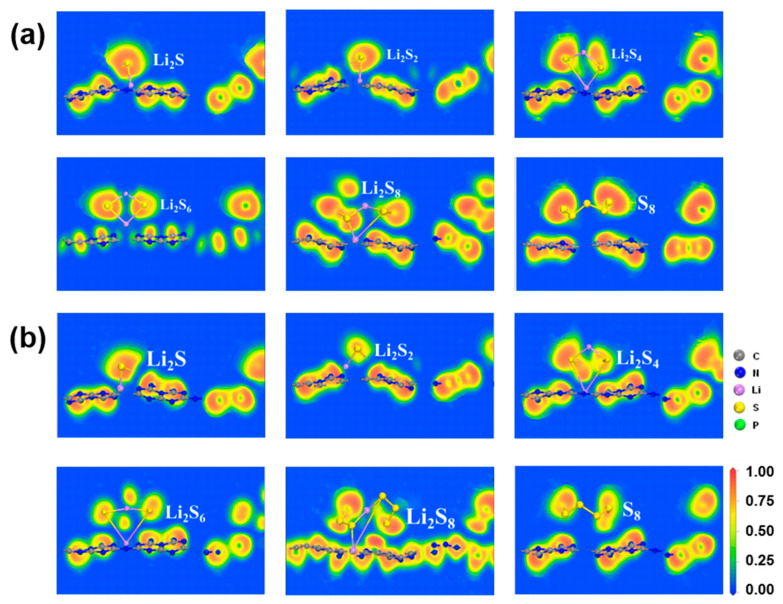
The electron localization function (ELF) plots of S_8_ and Li_2_S_n_ (n = 1, 2, 4, 6, 8) molecules anchored on the (**a**) g-C_3_N_4_ and (**b**) P_C_ substrates with the PBE functional.

**Figure 6:**
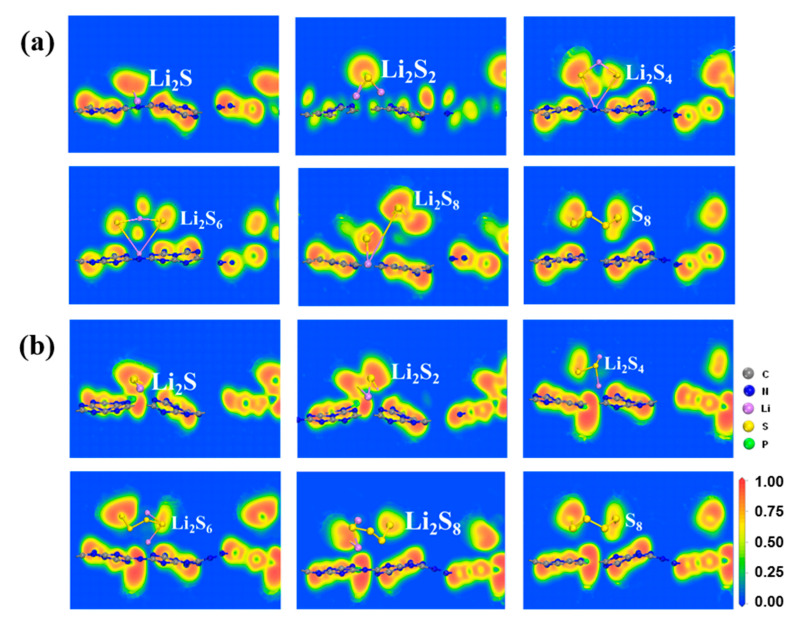
The electron localization function (ELF) plots of S_8_ and Li_2_S_n_ (n = 1, 2, 4, 6, 8) adsorbed on (**a**) P_N_ and (**b**) P_i_ substrates with the PBE functional.

However, the S_8_ molecule retains the puckered ring because of the negligible charge transfer between the S_8_ molecule and the substrate. Conversely, as illustrated in Figure 3 and Figure 4, and Figure S4 of SI, stronger Li-N bonds are discovered between the LiPSs and three P-doped substrates (P_C_, P_N_, and P_i_) in comparison with the counterpart of the g-C_3_N_4_ substrate. The strength of the Li-N bond within the LiPSs and the g-C_3_N_4_, P_C_, P_N_, and P_i_ substrates is further assessed by the electron localization function (ELF) [57,59], as depicted in Figure 5 and Figure 6 (P_N_ and P_i_ substrates). In general, ELF values of 0 and 1 denote the total electron localization and depletion, respectively. In contrast, the ELF values in the ranges of 0–0.5, 0.5–0.75, and 0.75–1 correspond to the ionic bond, metal bond, and covalent bond, respectively. The ELF values surrounding Li and N atoms are located within the range of 0.75–1, representing a covalent bond between the LiPSs cluster and the substrates. Compared with g-C_3_N_4_, the P_C_ substrate exhibits stronger Li-N bonds, which are beneficial for suppressing the shuttling of high-order Li_2_S_n_ (n = 4, 6, and 8) molecules.

**Figure 7:**
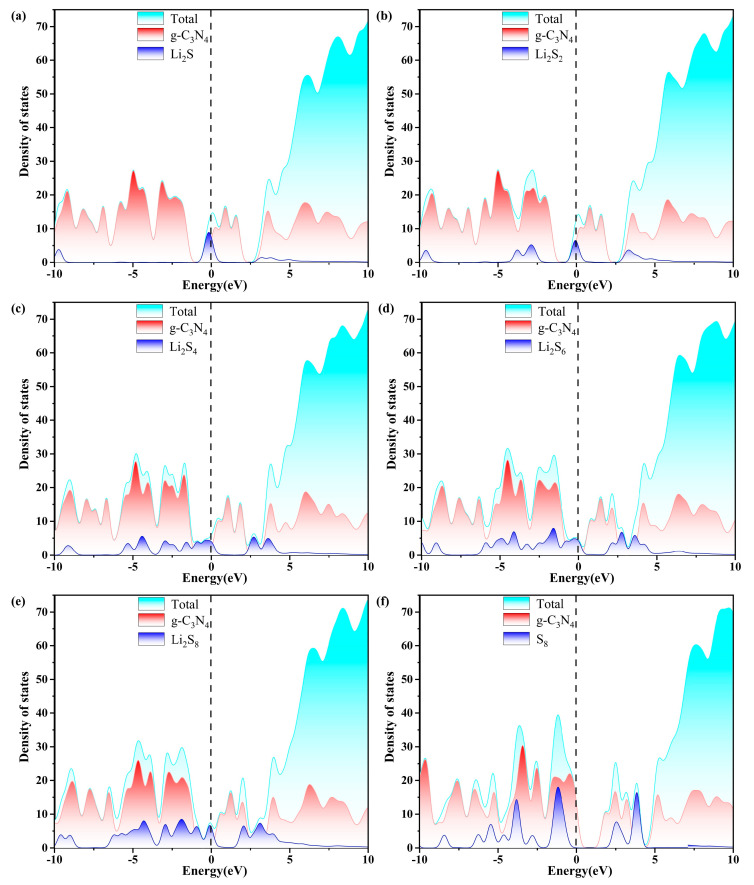
The calculated density of states (DOS) of (a–f) S_8_@g-C_3_N_4_ and LiPSs@g-C_3_N_4_ molecules. The dashed line indicates the Fermi level with the PBE functional.

**Figure 8:**
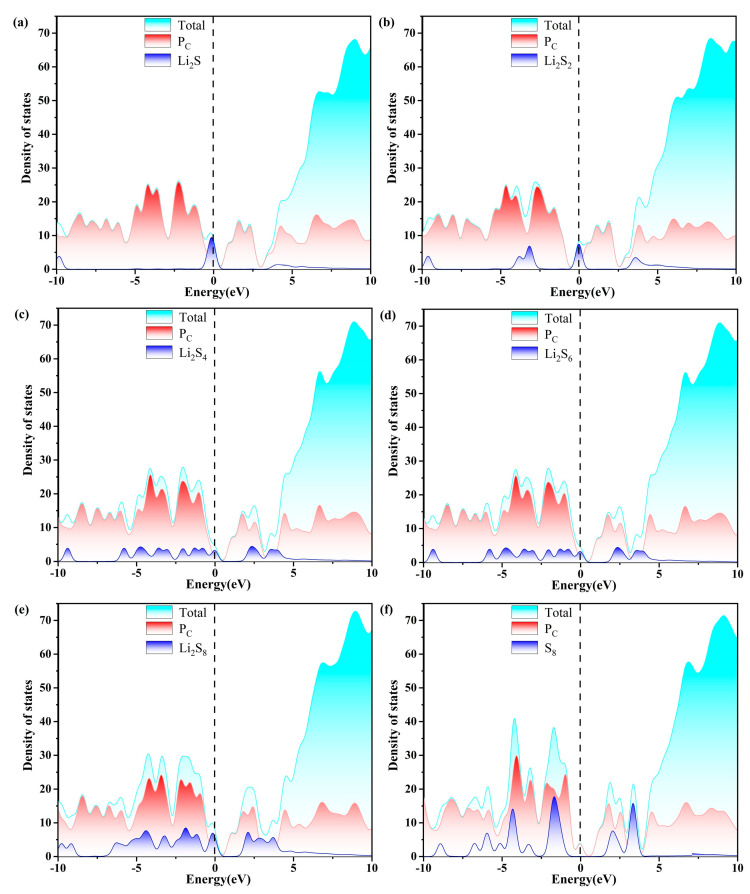
The calculated density of states (DOS) of (a–f) S_8_@P_C_ and LiPSs@P_C_ molecules. The dashed line indicates the Fermi level with the PBE functional.

**Figure 9:**
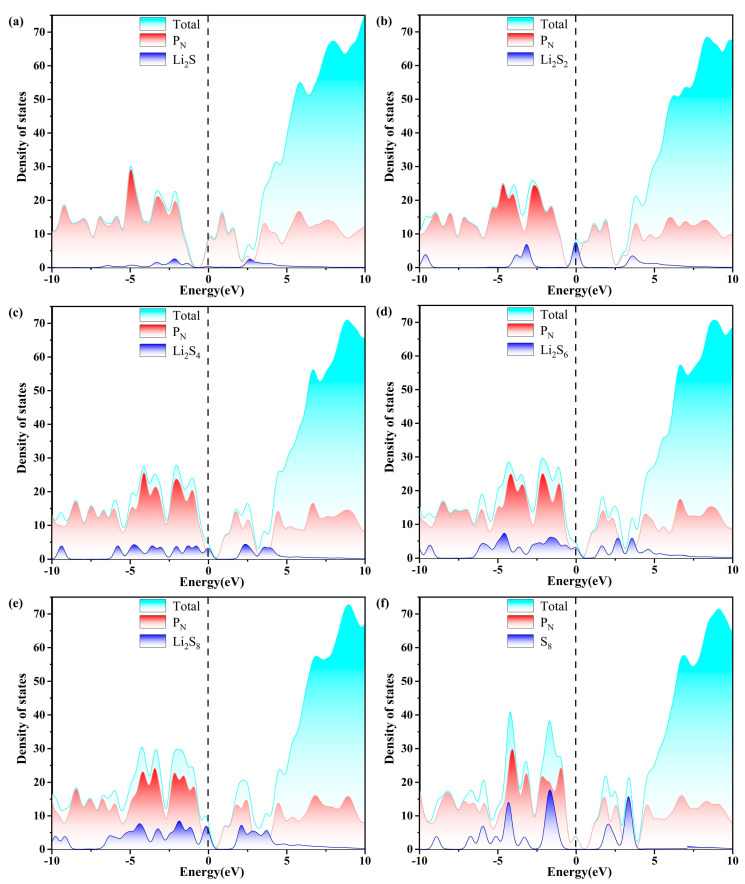
The calculated density of states (DOS) of (a–f)S_8_@P_N_ and LiPSs@P_N_ molecules. The dashed line indicates the Fermi level with the PBE functional.

**Figure 10:**
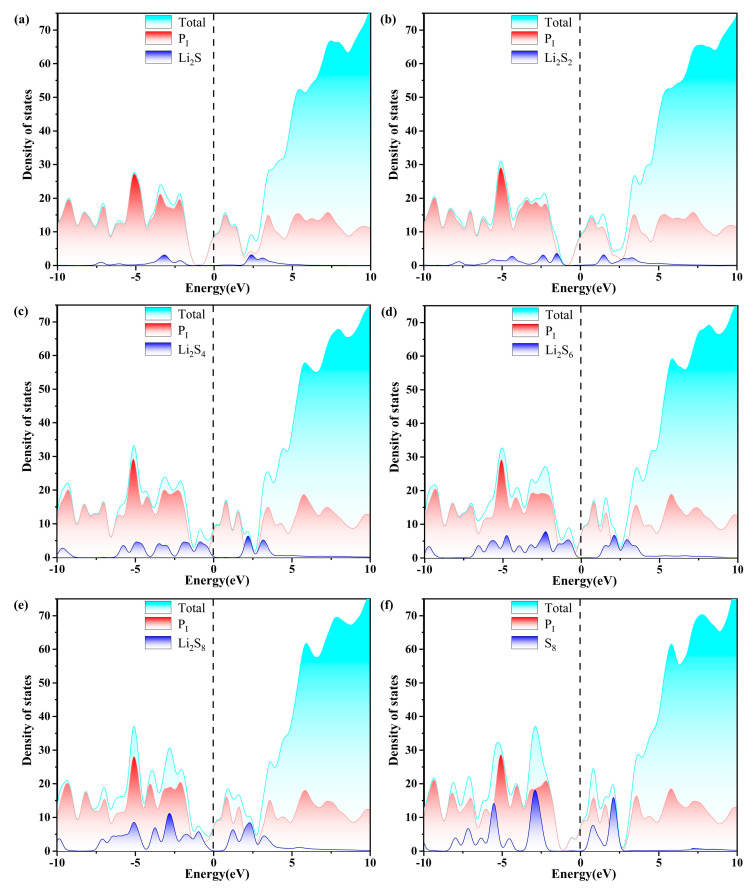
The calculated density of states (DOS) of (a–f) S_8_@P_i_ and LiPSs@P_i_ molecules. The dashed line indicates the Fermi level with the PBE functional.

Excellent conductivity is a crucial requirement for the host material of a Li-S battery with high performance. Consequently, the density of states (DOS) of S_8_ and Li_2_S_n_ (n = 1, 2, 4, 6, 8) molecules anchored on g-C_3_N_4_, P_C_, P_N_, and P_i_ substrates are evaluated, as plotted in Figure 7, Figure 8, Figure 9 and Figure 10. The pristine substrates exhibit semi-metallic properties. When the LiPSs molecule is anchored on the P-doping g-C_3_N_4_ monolayer, significant electronic states are observed near the Fermi level, rendering the metallic characteristics of the Li_2_S_n_@P_C_ system. The LiPSs molecules significantly contribute to the overall DOS, as revealed by the comprehensive PDOS investigation in the vicinity of the Fermi level. Consequently, P-g-C_3_N_4_ substrates are good candidates for host materials with high conductivity in Li-S batteries.

As depicted in Figure 7, Figure 8, Figure 9 and Figure 10, the band gap of S_8_ and the LiPSs upon anchoring on the surfaces of g-C_3_N_4_ and P-g-C_3_N_4_ are considerably smaller than the counterparts of pristine substrates. For the LiPSs@g-C_3_N_4_ or LiPSs@P-g-C_3_N_4_ systems, particularly for Li_2_S_4_ and Li_2_S_6_, electron distribution is observed near the Fermi level, indicating an increase in electrical conductivity. Further DOS analysis highlights the P_C_ substrate’s strong electrical conductivity, facilitating the Li-S battery’s electrochemical performance.

### 2.5. Energy Profiles and Decomposition of Li_2_S on the Surface of the Monolayer Substrates

**Figure 11:**
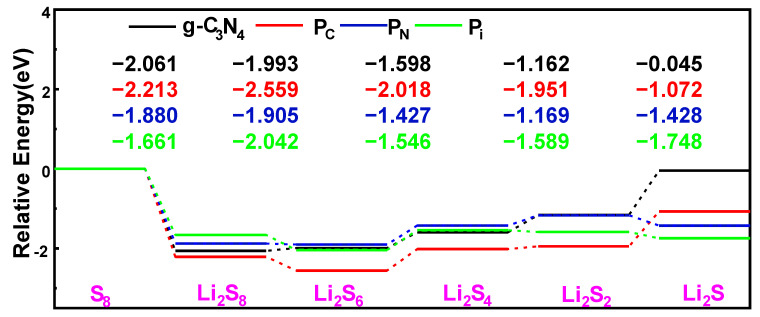
Energy profiles for reducing S_8_ and Li_2_S_n_ (n = 1, 2, 4, 6, 8) on the g-C_3_N_4_, P_C_, P_N_, and P_i_ substrates with the PBE functional.

**Figure 12:**
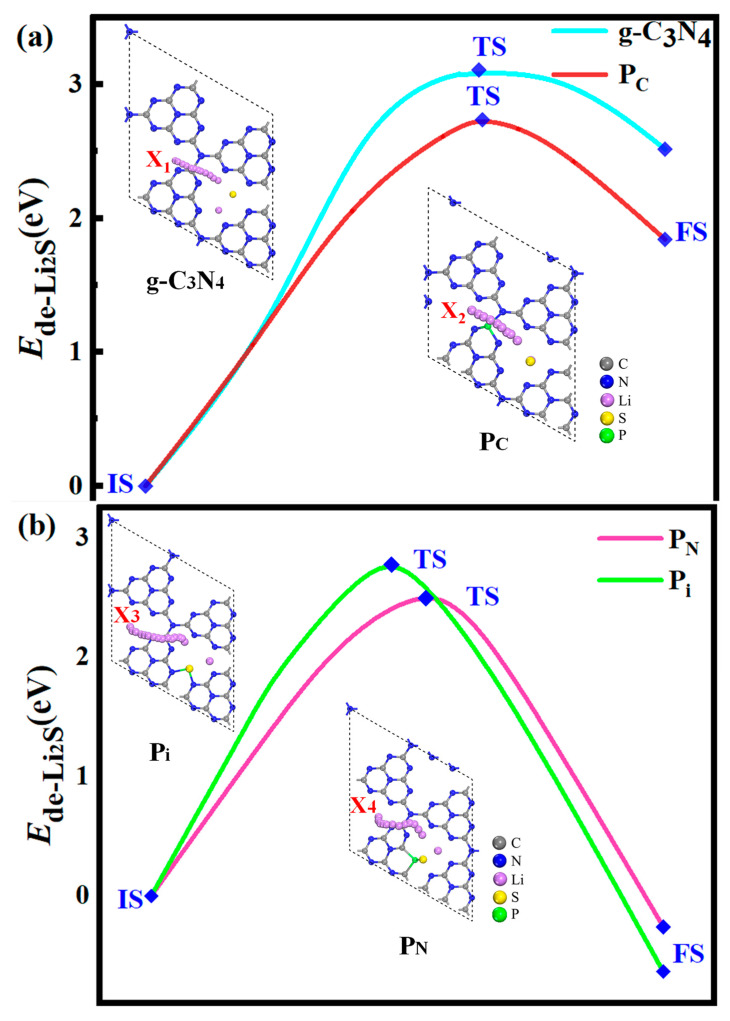
The energy barriers for the decomposition of Li_2_S on the g-C_3_N_4_ (**a**), P_C_ (**a**), P_N_ (**b**), and P_i_ (**b**) substrates with the PBE functional. Li_2_S undergoes three stages in the decomposition pathway: the initial state (IS), the transition state (TS), and the final state (FS). The top view depicts the decomposition path of the Li-ion from the IS to the FS.

To evaluate the catalytic behavior of P-g-C_3_N_4_ substrates, we focus on the energy profile distribution along the S_8_ reduction pathway and the decomposition of the Li_2_S molecule, as depicted in Figure 11 and Figure 12. These calculations are performed using the same parameter settings as in the structural optimization: PBE–Grimme generalized-ultrasoft pseudopotentials, K-points 3 × 3 × 1, and a cutoff energy of 550 eV. It is crucial to note that the choice of substrate plays a significant role in these processes. We observe similar reversible production processes of the S_8_ molecules for the g-C_3_N_4_, P_C_, P_N_, and P_i_ substrates. This involves the sequential reduction of the first two Li atoms and S_8_ to form Li_2_S_8_, followed by subsequent reduction and oxidation yielding Li_2_S_6_, Li_2_S_4_, and Li_2_S_2_ molecules, ultimately resulting in the final product Li_2_S. The S_8_ undergoes spontaneous exothermic conversion to Li_2_S_8_ and Li_2_S_6_. However, with the further decrease in S content, the energy changes of the subsequent formation of Li_2_S_4_ exhibit slightly different trends, and the transfer process becomes endothermic. Notably, endothermic reactions occur on g-C_3_N_4_ and P_C_ substrates, resulting in the Li_2_S formation. Meanwhile, the transfer processes of generating Li_2_S on the P_N_ and P_i_ substrates become exothermic. Li_2_S undergoes conversion to S_8_ through the Li-S bond cleavage during the charging process.

To evaluate the catalytic performance of the g-C_3_N_4_, P_C_, P_N_, and P_i_ substrates for the conversion of S active material, the decomposition of Li_2_S is assessed as a representative example. Li_2_S is selected because of the poor kinetic performance associated with the solid phase and the low utilization rate of S active material. The kinetics of Li_2_S oxidation is profoundly influenced by the initial stages of the charging process in Li-S batteries, namely, the decomposition of Li_2_S → LiS^−^ + Li^+^. To evaluate the decomposition barrier, the linear simultaneous transit/quadratic simultaneous-transit (LST/QST) approach is used [60] with the PBE functional, and a 2 × 2 × 1 Monkhorst–Pack *k*-point to identify the transition states. As shown in Figure 12a, the energy barrier for Li-S bond cleavage within Li_2_S along the X_1_ route of the g-C_3_N_4_ monolayer is 3.11 eV. The decomposition barrier for the X_2_ path on the Pc monolayer is 2.74 eV. Besides, the initial state (IS) energy is lower than the final state (FS) energy, demonstrating an exothermic reaction. At the same time, the IS energy is higher than the FS energy, showing an endothermic response in Figure 12b. However, the decomposition along other substrates (P_i_ and P_N_) reveals distinct features. Specifically, the favorable decomposition barrier for the P_i_ is 2.86 eV, whereas that of P_N_ substrates is 2.55 eV (Figure 12b). The barrier height is responsible for the reaction rate. Therefore, the decomposition barriers of the P-g-C_3_N_4_ substrates are considerably lower than the counterparts of the g-C_3_N_4_, suggesting that P-g-C_3_N_4_ substrates can effectively facilitate the decomposition of Li_2_S with decreasing decomposition barriers, which is beneficial for enhancing the utilization of sulfur-active material in the Li-S battery.

## 3. Computational Methods

First-principles calculations were performed with the CASTEP module implemented in the Materials Studio [61,62]. The Perdew–Burke–Ernzerhof (PBE) functional within the generalized gradient approximation (GGA) and Vanderbilt ultrasoft pseudopotentials were adopted [63,64]. Considering the van der Waals interaction between the substrates (g-C_3_N_4_ and P-g-C_3_N_4_) and the LiPSs, Grimme’s (DFT-D2) semi-empirical dispersion energy correction was incorporated into the first-principles calculations [65]. The Broyden–Fletcher–Goldfarb–Shanno (BFGS) algorithm was employed to facilitate structure optimization. Pseudo-atomic calculations were conducted for C, N, and Li with electron configurations of 2s^2^2p^2^, 2s^2^2p^3^, and 1s^2^2s^1^, respectively [66]. A cutoff energy of 550 eV and Monkhorst–Pack k-points of 3 × 3 × 1 were utilized. For the electronic structure calculations, a 6 × 6 × 1 mesh was adopted for k-point sampling. Considering the drawback of the GGA method in evaluating the electronic band structure of P-g-C_3_N_4_, the Heyd–Scuseria–Ernzerhof (HSE06) hybrid functional was employed [67,68]. A vacuum layer of 15 Å was incorporated along the z-axis to prevent interactions between adjacent g-C_3_N_4_ layers. The convergence criteria for energy, maximum force, and maximum displacement were 5.0 × 10^−6^ eV/atom, 0.01 eV/Å, and 5.0 × 10^−4^ Å, respectively. For the electronic structure calculations, the convergence criterion for energy was 1.0 × 10^−6^ eV/atom.

## 4. Conclusions

In this paper, the feasibility of using graphitic nitrogen carbide (g-C_3_N_4_) and phosphorus-doping substrate (P-g-C_3_N_4_) as anchoring materials for the Li-S battery was systematically investigated using first-principles calculations. Three moderately doped configurations were identified by calculating the formation energy of P-doping g-C3N4, considering the substitutional and interstitial sites: P_C_, P_N_, and P_i_. The PDOS results showed that the 2p of N was affected by introducing the P atom, and a synergistic effect occurred to enhance the energy bands of the P-g-C_3_N_4_ substrates. The energy band of P_C_ was elevated to 0.12 eV, that of P_i_ was 0.20 eV, and that of P_N_ was 2.14 eV; the P-g-C_3_N_4_ substrate showed metallicity, and the P-doping strategy enhanced the conductivity of the g-C_3_N_4_ monolayers. In addition, the substrate adsorption energy of P-g-C_3_N_4_ was enhanced by more than 40% compared to g-C_3_N_4_ due to the formation of robust Li-N bonds and the higher adsorption energies of S_8_ and Li_2_S_n_ (*n* = 1, 2, 4, 6, and 8). The Mulliken charge analysis showed that the electrons from the LiPSs molecules to the P-doped g-C_3_N_4_ substrate, especially the charge transfer of the long-chain LiPSs on the P_C_ substrate, increased by more than 1.5-fold. This means that the strong chemical interactions between them are formed and suppress the shuttling effect of higher-order LiPSs. Further density of states analyses showed that a transition from semi-metallic to metallic occurred in the P-doping g-C_3_N_4_ monolayers, which is favorable for electrochemical performance. Furthermore, the energy distribution and decomposition barriers of Li_2_S and P-doping on the g-C_3_N_4_ monolayers had similar reaction mechanisms and catalytic properties. In addition, the decomposition barriers of the Li_2_S molecules on P-doping g-C_3_N_4_ were in the range of 2.55–2.86 eV, which was lower than that on g-C_3_N_4_ (3.11 eV). Therefore, the P-g-C_3_N_4_ substrates significantly improve Li_2_S formation and decomposition, thus increasing the utilization and fast reaction kinetics of the sulfur in the Li-S battery. This work provides detailed theoretical guidance for the experimental synthesis of doped elements as catalysts, and the application of P-g-C_3_N_4_ can be used as a promising anchoring material for a high-performance Li-S battery.

## Figures and Tables

**Table 1:** Comparison of lattice parameters of g-C_3_N_4_ with various theoretical methods.

Method	vdW Correction	a/Å	b/Å
LDA-CAPZ	no	7.072	7.072
GGA-PBE	no	7.144	7.144
GGA-PBE	Grimme	7.144	7.144
GGA-PBE	TS	7.138	7.138
Exp. ^a^	-	7.30	-
Other theory ^b^	OBS	7.16	-
Other theory ^c^	-	7.13	-

^a^ Bojdys et al. (ref. [32]), ^b^ Ma et al. (ref. [38]), ^c^ Gracia et al. (ref. [36]).

**Table 2:** The formation energies (*E**_f_,*** eV) of the P-doping g-C_3_N_4_ monolayer at different doping positions with the PBE functional.

P-Doping Positions	C1	C2	C3	C4	N1	N2	N3	N4	Interstitial (P_i_)
*E_f_* (eV) (2 × 2 × 1)	1.06	1.81	1.07	1.78	2.31	1.34	1.34	5.03	0.81
*E_f_* (eV) (4 × 4 × 1)	0.77	1.65	0.78	1.62	1.70	1.20	1.20	4.83	0.80
*E_f_* (eV) ^a^	0.73	1.52	-	-	1.93	1.33	-	3.55	0.78

^a^ Ma et al. (ref. [38]).

**Table 3:** Band gaps of the g-C_3_N_4_ monolayer and P-g-C_3_N_4_ (P_C_, P_N_, and P_i_) with the HSE06 functional.

*E_g_* Gap (eV)	g-C_3_N_4_	P Substitute N Site (P_N_)	P Substitute C Site (P_C_)	P-Doping Interstitial Site (P_i_)
GGA-PBE	1.13	0.89	0.04	0.14
HSE06	2.67	2.14	0.12	0.20
Exp ^a^	2.70	-	-	-

^a^ Wang et al. (ref. [46]).

**Table 4:** The average S-S, Li-S, and Li-N distances (Å) and adsorption energy (eV) of the S_8_ and LiPSs anchoring on the g-C_3_N_4_ and P_C_ substrates.

Molecules	LiPSs		g-C_3_N_4_			P_C_		
	S-S	Li-S	Li-S	Li-N	*E_ad_*	Li-S	Li-N	*E_ad_*
Li_2_S	-	2.108	2.264	2.400	3.809	2.342	1.992	6.760
Li_2_S_2_	2.186	2.240	2.593	2.397	3.773	2.515	1.965	6.525
Li_2_S_4_	2.093	2.386	3.040	2.351	3.350	2.336	2.250	5.225
Li_2_S_6_	2.078	2.428	2.531	2.069	3.198	2.411	2.280	5.245
Li_2_S_8_	2.075	2.419	2.781	2.065	3.392	2.473	2.299	5.026
S_8_	2.060	-	-	-	2.021	-	-	3.502

## Data Availability

The data supporting this study’s findings are available from the corresponding author upon reasonable request.

## Supplementary Materials

The following supporting information can be downloaded at https://www.mdpi.com/article/10.3390/molecules29122746/s1: Figure S1: Structures of (a) S_8_ and LiPSs. (b) g-C_3_N_4_. (c) P-doped configurations of g-C_3_N_4_; Figure S2: The calculated band structure of the four substrates: (a) g-C_3_N_4_, (b) P_N_, (c) P_C_, and (d) P_i_, with the HSE06 functional; Figure S3: The projected density of states (PDOS) of (a) g-C_3_N_4_. (b) P_N_, (c) P_C_, and (d) P_i_; Figure S4: Charge transfers of Li_2_S_n_ (n = 1, 2, 4, 6 and 8) and S_8_ clusters adsorbed on P_N_ (a) and P_i_ (b) substrates; Table S1: Top views of optimization configurations for the most stable adsorption of S_8_ and Li_2_S_n_ (n = 1, 2, 4, 6, 8) molecules on the four different monolayer substrates; Table S2: Adsorption energies (E_ad_) of S_8_ and Li_2_S_n_ (n = 1, 2, 4, 6, 8) at lithiation stages on the difference substrates; Table S3: The average distance of LiPSs adsorption on P_N_ and P_i_ substrate materials; and Table S4: The Hirshfield charge analysis of S_8_ and Li_2_S_n_ (n = 1, 2, 4, 6, 8) adsorbed on the different substrates.

## Abbreviations

The following abbreviations are used in this manuscript:

DFT	Density functional theory
GGA	Generalized gradient approximation
LDA-CAPZ	Local density approximation of Ceperley and Alder by Perdew and Zunger
TS	Tkatchenko-Scheffler
vdW	van der Waals
BFGS	Broyden–Fletcher–Goldfarb–Shanno
PDOS	Partial density of states
ELF	Electron localization function
LST/QST	Linear simultaneous transit/quadratic simultaneous transit

## References

[1] Xu R., Lu J., Amine K. (2015). Progress in Mechanistic Understanding and Characterization Techniques of Li-S Batteries. Adv. Energy Mater..

[2] Rosenman A., Markevich E., Salitra G., Aurbach D., Garsuch A., Chesneau F. (2015). Review on Li-Sulfur Battery Systems: An Integral Perspective. Adv. Energy Mater..

[3] Wild M., O’neill L., Zhang T., Purkayastha R., Minton G., Marinescu M., Offer G. (2015). Lithium sulfur batteries, a mechanistic review. Energy Environ. Sci..

[4] Ogoke O., Wu G., Wang X., Casimir A., Ma L., Wu T., Lu J. (2017). Effective strategies for stabilizing sulfur for advanced lithium-sulfur batteries. J. Mater. Chem. A.

[5] Wang T., Dong Q., Li C., Wei Z. (2024). Sulfur Cathode Electrocatalysis in Lithium-Sulfur Batteries: A Comprehensive Understanding. Acta Phys. Chim. Sin..

[6] Fang R., Chen K., Yin L., Sun Z., Li F., Cheng H. (2019). The Regulating Role of Carbon Nanotubes and Graphene in Lithium-Ion and Lithium–Sulfur Batteries. Adv. Mater..

[7] Liu L., Zheng Y., Sun Y., Pan H. (2024). Modulation of Potential-Limiting Steps in Lithium-Sulfur Batteries by Catalyst Synergy. Small.

[8] Pei F., Lin L.L., Fu A., Mo S.G., Ou D.H., Fang X.L., Zheng N.F. (2018). A Two-Dimensional Porous Carbon-Modified Separator for High-Energy-Density Li-S Batteries. Joule.

[9] Qin X.Y., Wu J.X., Xu Z.L., Chong W.G., Huang J.Q., Liang G.M., Li B.H., Kang F.Y., Kim J.K. (2019). Electrosprayed multiscale porous carbon microspheres as sulfur hosts for long-life lithium-sulfur batteries. Carbon.

[10] Zhao C., Xu G.L., Yu Z., Zhang L.C., Hwang I., Mo Y.X., Ren Y.X., Cheng L., Sun C.J., Ren Y. (2021). A high-energy and long-cycling lithium-sulfur pouch cell via a macroporous catalytic cathode with double-end binding sites. Nat. Nanotechnol..

[11] Zheng B., Lin X., Zhang X., Wu D., Matyjaszewski K. (2020). Emerging functional porous polymeric and carbonaceous materials for environmental treatment and energy storage. Adv. Funct. Mater..

[12] Li H., Liu D., Zhu X.X., Qu D.Y., Xie Z.Z., Li J.S., Tang H.L., Zheng D., Qu D.Y. (2020). Integrated 3D electrodes based on metal-nitrogen-doped graphitic ordered mesoporous carbon and carbon paper for high-loading lithium-sulfur batteries. Nano Energy.

[13] He M.X., Li X., Li W.H., Zheng M.T., Wang J.J., Ma S.B., Ma Y.L., Yin G.P., Zuo P.J., Sun X.L. (2021). Immobilization and kinetic promotion of polysulfides by molybdenum carbide in lithium-sulfur batteries. Chem. Eng. J..

[14] Jo S.C., Hong J.W., Choi I.H., Kim M.J., Kim B.G., Lee Y.J., Choi H.Y., Kim D., Kim T., Baeg K.J. (2022). Multimodal Capturing of Polysulfides by Phosphorus-Doped Carbon Composites for Flexible High-Energy-Density Lithium-Sulfur Batteries. Small.

[15] Zhong Y., Chao D.L., Deng S.J., Zhan J.Y., Fang R.Y., Xia Y., Wang Y.D., Wang X.L., Xia X.H., Tu J.P. (2018). Confining Sulfur in Integrated Composite Scaffold with Highly Porous Carbon Fibers/Vanadium Nitride Arrays for High-Performance Lithium-Sulfur Batteries. Adv. Funct. Mater..

[16] Park J., Yu B., Park J., Choi J., Kim C., Sung Y.E., Goodenough J. (2017). Tungsten Disulfide Catalysts Supported on a Carbon Cloth Interlayer for High-Performance Li-S Battery. Adv. Energy Mater..

[17] Wu S.Y., Li X., Zhang Y.Z., Guan Q.H., Wang J., Shen C.Y., Lin H.Z., Wang J.T., Wang Y.L., Zhan L. (2023). Interface engineering of MXene-based heterostructures for lithium-sulfur batteries. Nano Res..

[18] He Y.B., Chang Z., Wu S.C., Qiao Y., Bai S.Y., Jiang K.Z., He P., Zhou H.S. (2018). Simultaneously Inhibiting Lithium Dendrites Growth and Polysulfides Shuttle by a Flexible MOF-Based Membrane in Li-S Batteries. Adv. Energy Mater..

[19] Haseeb H.H., Li Y., Ayub S., Fang Q.L., Yu L.J., Xu K.W., Ma F. (2020). Defective Phosphorene as a Promising Anchoring Material for Lithium-Sulfur Batteries. J. Phys. Chem. C.

[20] Zheng Y.P., Li H.H., Yuan H.Y., Fan H.H., Li W.L., Zhang J.P. (2018). Understanding the anchoring effect of Graphene, BN, CN and CN monolayers for lithium-polysulfides in Li-S batteries. Appl. Surf. Sci..

[21] Du M.J., Tian X.Q., Ran R., Zhou W., Liao K.M., Shao Z.P. (2020). Tuning Nitrogen in Graphitic Carbon Nitride Enabling Enhanced Performance for Polysulfide Confinement in Li-S Batteries. Energy Fuels.

[22] Li D.S., Liu J., Wang W.J., Li S.M., Yang G.L., Wang P., Zhu K.X., Li Z.J. (2021). Synthesis of porous N deficient graphitic carbon nitride and utilization in lithium-sulfur battery. Appl. Surf. Sci..

[23] Tong Z.M., Huang L., Liu H.P., Lei W., Zhang H.J., Zhang S.W., Jia Q.L. (2021). Defective Graphitic Carbon Nitride Modified Separators with Efficient Polysulfide Traps and Catalytic Sites for Fast and Reliable Sulfur Electrochemistry. Adv. Funct. Mater..

[24] Sun W.H., Song Z.H., Feng Z.X., Huang Y.Q., Xu Z.J., Lu Y.C., Zou Q.L. (2022). Carbon-Nitride-Based Materials for Advanced Lithium-Sulfur Batteries. Nano-Micro Lett..

[25] Fina F., Callear S.K., Carins G.M., Irvine J.T.S. (2015). Structural Investigation of Graphitic Carbon Nitride via XRD and Neutron Diffraction. Chem. Mater..

[26] Jun Y.S., Hong W.H., Antonietti M., Thomas A. (2009). Mesoporous, 2D Hexagonal Carbon Nitride and Titanium Nitride/Carbon Composites. Adv. Mater..

[27] Li S.N., Yang S.B., Shen D., Sun W., Shan X.Y., Dong W., Chen Y.H., Zhang X., Mao Y.Q., Tang S.W. (2017). Polysulfide intercalation in bilayer-structured graphitic CN: A first-principles study. Phys. Chem. Chem. Phys..

[28] Thomas A., Fischer A., Goettmann F., Antonietti M., Müller J.-O., Schlögl R., Carlsson J.M. (2008). Graphitic carbon nitride materials: Variation of structure and morphology and their use as metal-free catalysts. J. Mater. Chem..

[29] Zhang Y., Mori T., Ye J., Antonietti M. (2010). Phosphorus-doped carbon nitride solid: Enhanced electrical conductivity and photocurrent generation. J. Am. Chem. Soc..

[30] Zhang X., Yang S.B., Chen Y.H., Li S.N., Tang S.W., Shen D., Dong W., Hao D.Y. (2020). Effect of phosphorous-doped graphitic carbon nitride on electrochemical properties of lithium-sulfur battery. Ionics.

[31] Ma T.Y., Ran J.R., Dai S., Jaroniec M., Qiao S.Z. (2015). Phosphorus-Doped Graphitic Carbon Nitrides Grown In Situ on Carbon-Fiber Paper: Flexible and Reversible Oxygen Electrodes. Angew. Chem. Int. Ed..

[32] Bojdys M.J., Müller J.O., Antonietti M., Thomas A. (2008). Ionothermal synthesis of crystalline, condensed, graphitic carbon nitride. Chem. Eur. J..

[33] Liang X., Hart C., Pang Q., Garsuch A., Weiss T., Nazar L.F. (2015). A highly efficient polysulfide mediator for lithium-sulfur batteries. Nat. Commun..

[34] Liu R.L., Wei Z.Y., Peng L.L., Zhang L.Y., Zohar A., Schoeppner R., Wang P.Q., Wan C.Z., Zhu D., Liu H.T. (2024). Establishing reaction networks in the 16-electron sulfur reduction reaction. Nature.

[35] Kang X.Y., He T.Q., Zou R., Niu S.T., Ma Y.X., Zhu F.L., Ran F. (2024). Size Effect for Inhibiting Polysulfides Shuttle in Lithium-Sulfur Batteries. Small.

[36] Gracia J., Kroll P. (2009). Corrugated layered heptazine-based carbon nitride: The lowest energy modifications of C_3_N_4_ ground state. J. Mater. Chem..

[37] Ma X., Wu Y., Lu Y., Xu J., Wang Y., Zhu Y. (2011). Effect of compensated codoping on the photoelectrochemical properties of anatase TiO_2_ photocatalyst. J. Phys. Chem. C.

[38] Ma X., Lv Y., Xu J., Liu Y., Zhang R., Zhu Y. (2012). A strategy of enhancing the photoactivity of g-C_3_N_4_ via doping of nonmetal elements: A first-principles study. J. Phys. Chem. C.

[39] Liu J.J. (2016). Effect of phosphorus doping on electronic structure and photocatalytic performance of g-CN: Insights from hybrid density functional calculation. J. Alloys Compd..

[40] Yang S., Gong Y., Zhang J., Zhan L., Ma L., Fang Z., Vajtai R., Wang X., Ajayan P.M. (2013). Exfoliated Graphitic Carbon Nitride Nanosheets as Efficient Catalysts for Hydrogen Evolution Under Visible Light. Adv. Mater..

[41] Ceperley D.M., Alder B.J. (1980). Ground State of the Electron Gas by a Stochastic Method. Phys. Rev. Lett..

[42] Perdew J.P., Zunger A. (1981). Self-interaction correction to density-functional approximations for many-electron systems. Phys. Rev. B.

[43] Pan Y., Li Q., Zhu Y., Li Y., Liu H., Cong Y., Wu M. (2023). Homonuclear transition-metal dimers embedded monolayer C_2_N as promising anchoring and electrocatalytic materials for lithium-sulfur battery: First-principles calculations. Appl. Surf. Sci..

[44] Yamsang N., Sittiwong J., Srifa P., Boekfa B., Sawangphruk M., Maihom T., Limtrakul J. (2021). First-Principle study of lithium polysulfide adsorption on heteroatom doped graphitic carbon nitride for Lithium-Sulfur batteries. Appl. Surf. Sci..

[45] Mosquera-Lois I., Kavanagh S.R., Klarbring J., Tolborg K., Walsh A. (2023). Imperfections are not 0 K: Free energy of point defects in crystals. Chem. Soc. Rev..

[46] Wang X., Maeda K., Thomas A., Takanabe K., Xin G., Carlsson J.M., Domen K., Antonietti M. (2009). A metal-free polymeric photocatalyst for hydrogen production from water under visible light. Nat. Mater..

[47] Mott N. (1987). The mobility edge since 1967. J. Phys. C Solid State Phys..

[48] Kundu A., Song Y., Galli G. (2022). Influence of nuclear quantum effects on the electronic properties of amorphous carbon. Proc. Natl. Acad. Sci. USA.

[49] Kundu A., Galli G. (2024). Quantum Vibronic Effects on the Excitation Energies of the Nitrogen-Vacancy Center in Diamond. J. Phys. Chem. Lett..

[50] Guo S., Tang Y., Xie Y., Tian C., Feng Q., Zhou W., Jiang B. (2017). P-doped tubular g-C_3_N_4_ with surface carbon defects: Universal synthesis and enhanced visible-light photocatalytic hydrogen production. Appl. Catal. B Environ..

[51] Liu S., Zhu H., Yao W., Chen K., Chen D. (2018). One step synthesis of P-doped g-C_3_N_4_ with the enhanced visible light photocatalytic activity. Appl. Surf. Sci..

[52] Chen Y.S., Li S., Dong W., Shen D. (2018). Applications of first-principles in cathode materials of lithium-sulfide batteries. Chin. Nonf. Met..

[53] Dong W., Zhu X., Shen D., Zhao M., Gu H., Yang F., Chang Q., Tang S., Hong X., Yang S. (2023). Uncovering the lithium-embedded behavior and catalytic mechanism of g-C_3_N_4_ as a sulfur host of lithium-sulfur batteries in the initial discharge reaction. Diamond Relat. Mater..

[54] Lin H., Liu G., Zhu L., Zhang Z., Jin R., Huang Y., Gao S. (2021). Flexible borophosphene monolayer: A potential Dirac anode for high-performance non-lithium ion batteries. Appl. Surf. Sci..

[55] Mulliken R.S. (1955). Electronic population analysis on LCAO–MO molecular wave functions. I. J. Chem. Phys..

[56] Fonseca Guerra C., Handgraaf J.W., Baerends E.J., Bickelhaupt F.M. (2004). Voronoi deformation density (VDD) charges: Assessment of the Mulliken, Bader, Hirshfeld, Weinhold, and VDD methods for charge analysis. J. Comput. Chem..

[57] Becke A.D., Edgecombe K.E. (1990). A simple measure of electron localization in atomic and molecular systems. J. Chem. Phys..

[58] Martin F., Zipse H. (2004). Charge distribution in the water molecule—A comparison of methods. J. Comput. Chem..

[59] Silvi B., Savin A. (1994). Classification of chemical bonds based on topological analysis of electron localization functions. Nature.

[60] Govind N., Petersen M., Fitzgerald G., King-Smith D., Andzelm J. (2003). A generalized synchronous transit method for transition state location. Comput. Mater. Sci..

[61] Segall M., Lindan P.J., Probert M.A., Pickard C.J., Hasnip P.J., Clark S., Payne M. (2002). First-principles simulation: Ideas, illustrations and the CASTEP code. J. Phys. Condens. Matter.

[62] Clark S.J., Segall M.D., Pickard C.J., Hasnip P.J., Probert M.I.J., Refson K., Payne M.C. (2005). First principles methods using CASTEP. Z. Für Krist. Cryst. Mater..

[63] Vanderbilt D. (1990). Soft self-consistent pseudopotentials in a generalized eigenvalue formalism. Phys. Rev. B.

[64] Perdew J.P., Burke K., Ernzerhof M. (1996). Generalized gradient approximation made simple. Phys. Rev. Lett..

[65] Grimme S. (2006). Semiempirical GGA-type density functional constructed with a long-range dispersion correction. J. Comput. Chem..

[66] Fischer T.H., Almlof J. (1992). General methods for geometry and wave function optimization. J. Phys. Chem. C.

[67] Krukau A.V., Vydrov O.A., Izmaylov A.F., Scuseria G.E. (2006). Influence of the exchange screening parameter on the performance of screened hybrid functionals. J. Chem. Phys..

[68] Cohen A.J., Mori-Sánchez P., Yang W. (2008). Insights into current limitations of density functional theory. Science.

