# Supplementary material for: First-Principles Investigation of Phosphorus-Doped Graphitic Carbon Nitride as Anchoring Material for the Lithium-Sulfur Battery
Source: Molecules. 2024 Jun 9;29(12):2746. doi: 10.3390/molecules29122746 (PMC11206158; doi:10.3390/molecules29122746)
Supplement: Supplementary file 1 [file molecules-29-02746-s001.zip › molecules-3032788-supplementary.pdf]

Supporting Information

Contents

1. Figure S1. Structures of (a) S_8 and LiPSs. (b) g-C_3N_4. (c) P doped configurations g-C_3N_4……….……….………….……….……Page S1

2. Figure S2. The calculated Band structure of the four substrates: (a) g-C_3N_4, (b) P_N, (c) P_C, (d) P_i ……………………………Page S2

3. Figure S3. The projected density of states (PDOS) of (a) g-C_3N_4, (b) P_N, (c) P_C, (d) P_i...………...………………...…...…...…...….... Page S3

4. Table S1. Top views of optimization configurations for the most stable adsorption of S_8 and Li_2S_n on the substrates........Page S4

5. Table S2. Adsorption energies (E_{ad}) of S_8 and Li_2S_n (n = 1, 2, 4, 6, 8) at lithiation stages on the different substrates........Page S6

6. Table S3. The average distance of LiPSs adsorption on P_N and P_i substrate materials.……...…...…...…...…...…...…...Page S6

7. Figure S4. Charge transfers of Li_2S_n (n= 1, 2, 4, 6, and 8) and S_8 clusters adsorbed on P_N (a) and P_i (b) substrates.……... Page S7

8. Table S4 The Hirshfield charge analysis of S_8 and Li_2S_n (n = 1, 2, 4, 6, 8) adsorbed on the different substrates...Page S8

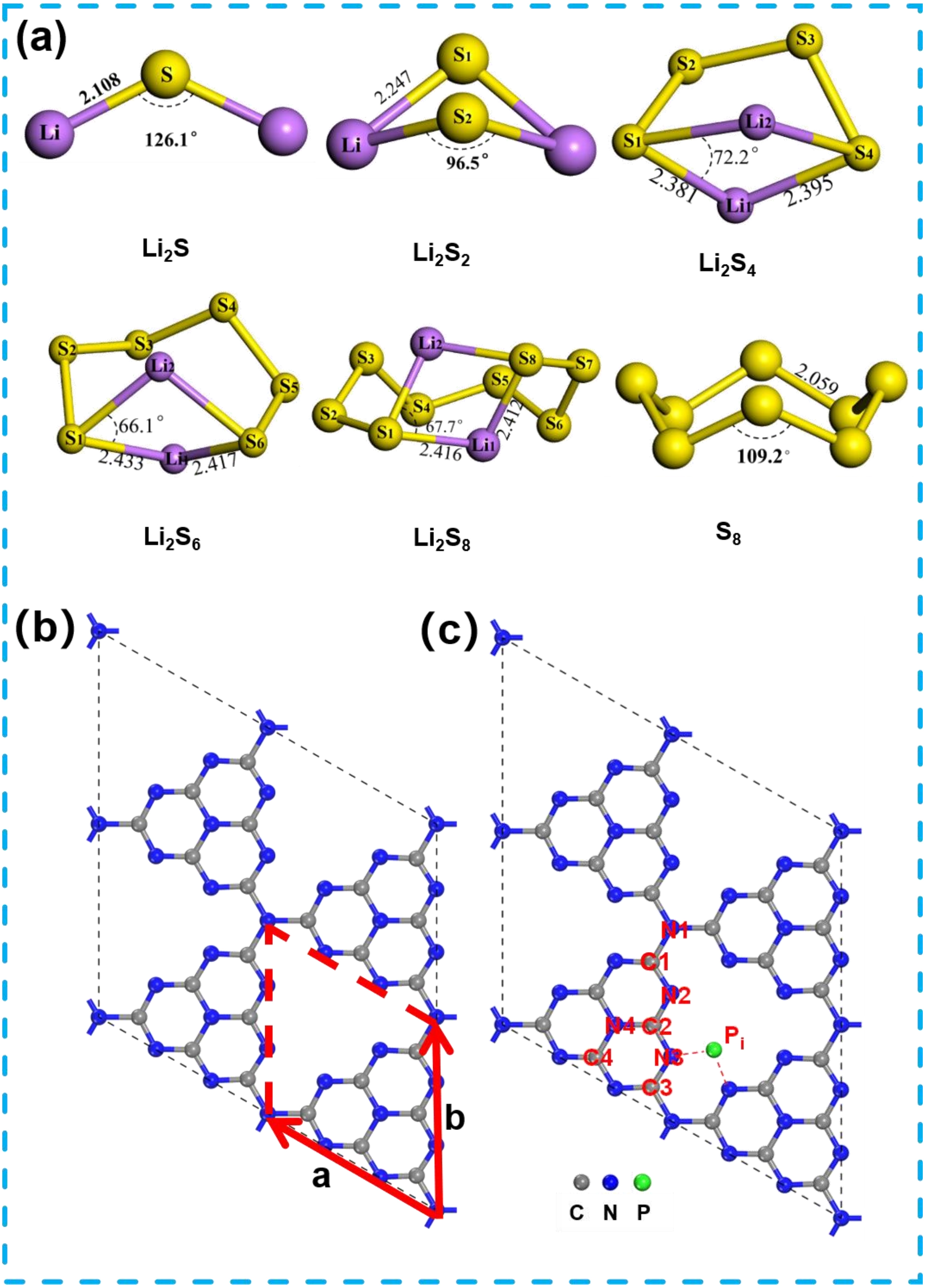

Figure S1. Structures of (a) S_8 and LiPSs. (b) g-C_3N_4. (c) P-doped configurations, including the substitutional and interstitial doped monolayer C_3N_4 with PBE functional. Roman numbers labeled on the C and N atoms identify the sites considered in this work.

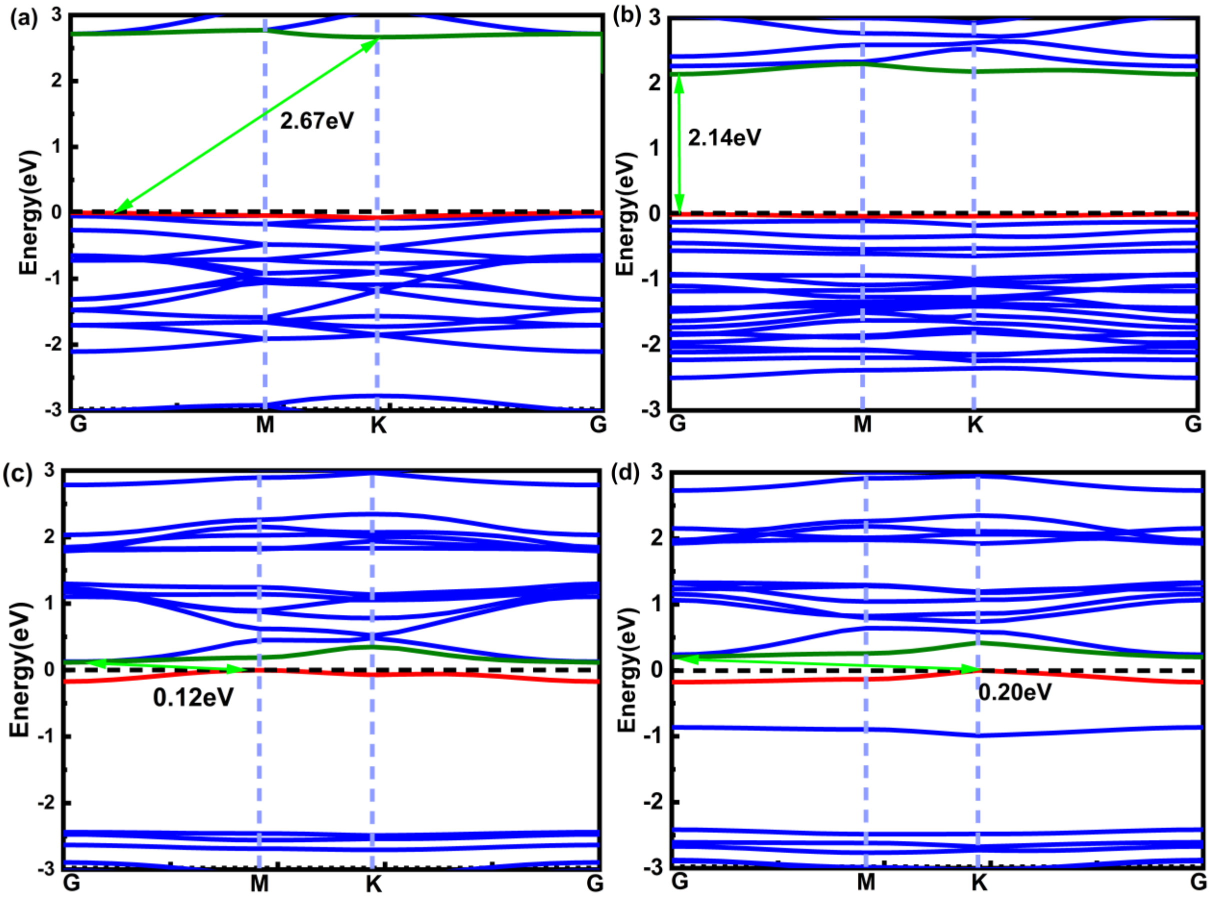

Figure S2. The calculated band structure of the four substrates: (a) g-C_3N_4, (b) P_N, (c) P_C, and (d) P_i with HSE06 functional. The Fermi level is set to zero energy and indicated by horizontal lines.

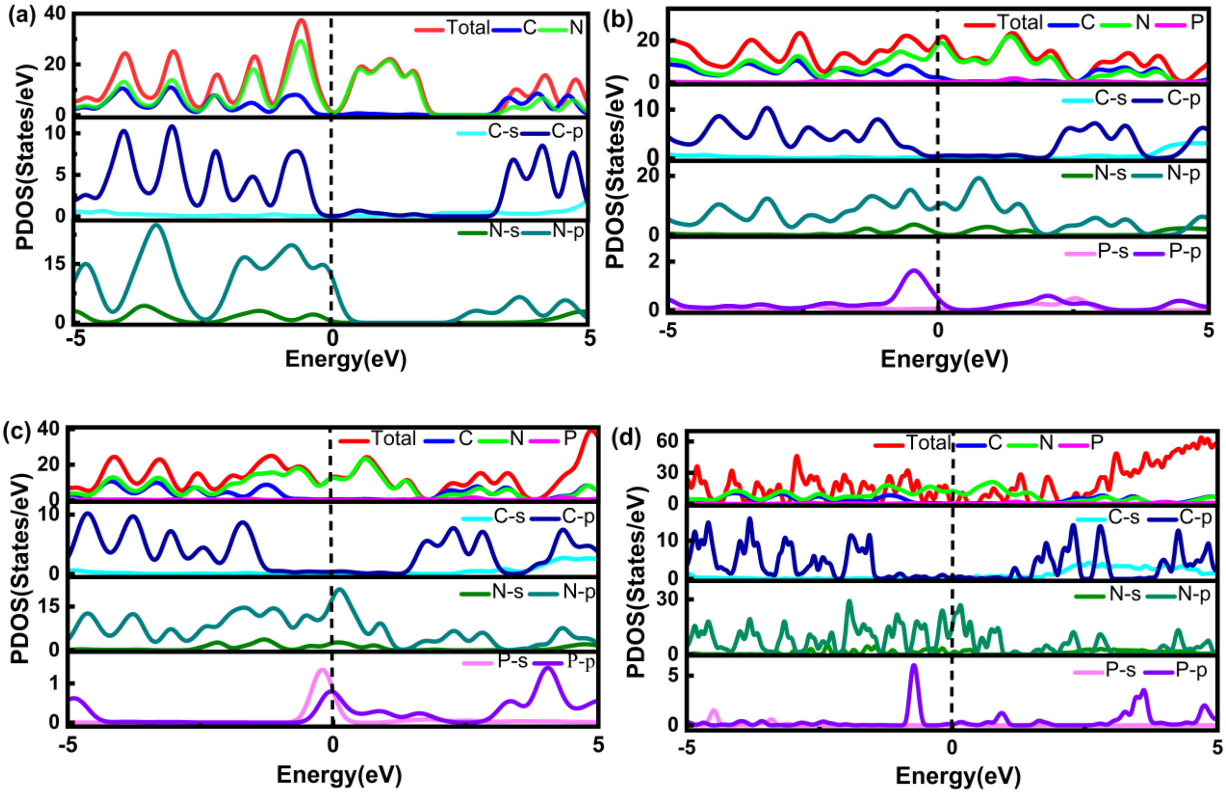

Figure S3. The projected density of states (PDOS) of (a) g-C_3N_4. (b) P_N, (c) P_C, (d) P_i with HSE06 functional. The Fermi level is set to zero.

Table S1. Top views of optimization configurations for the most stable adsorption of S_8 and Li_2S_n (n = 1, 2, 4, 6, 8) molecules on the four different monolayer substrates.

View / structures	Li_2S	Li_2S_2	Li_2S_4	Li_2S_6	Li_2S_8	S_8
g-C_3N_4	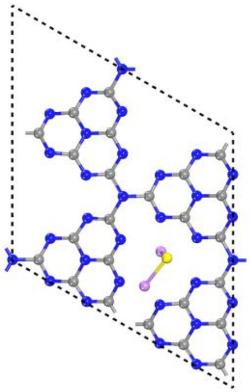	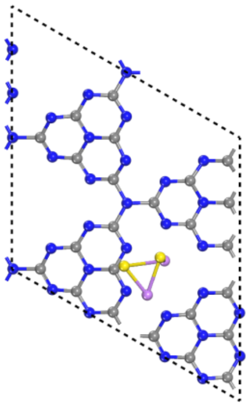	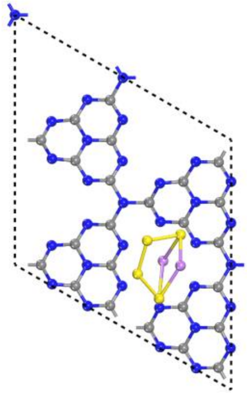	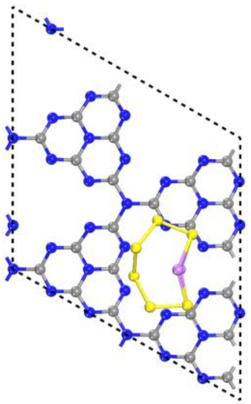	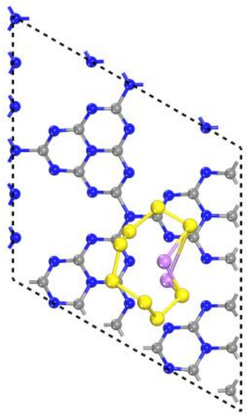	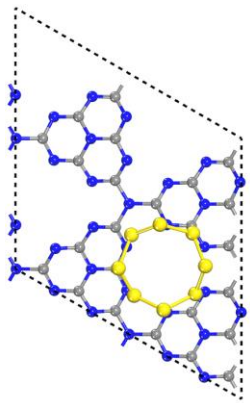
P_C	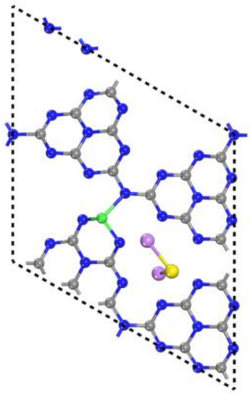	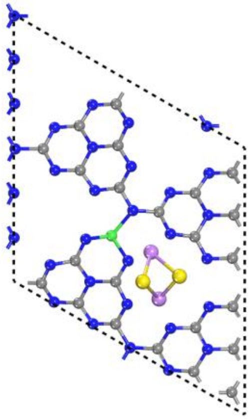	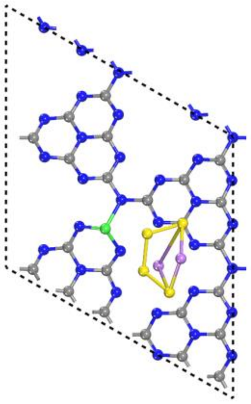	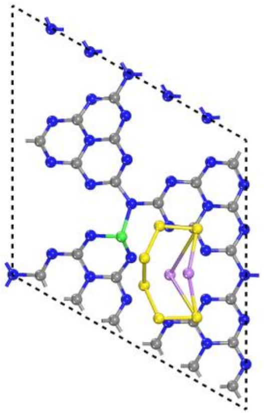	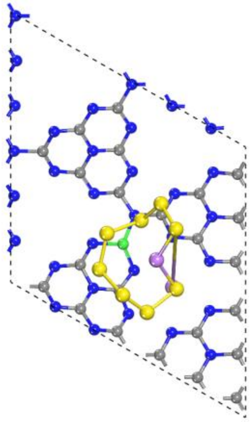	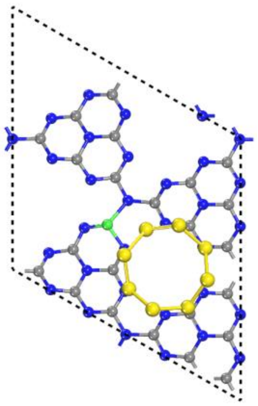

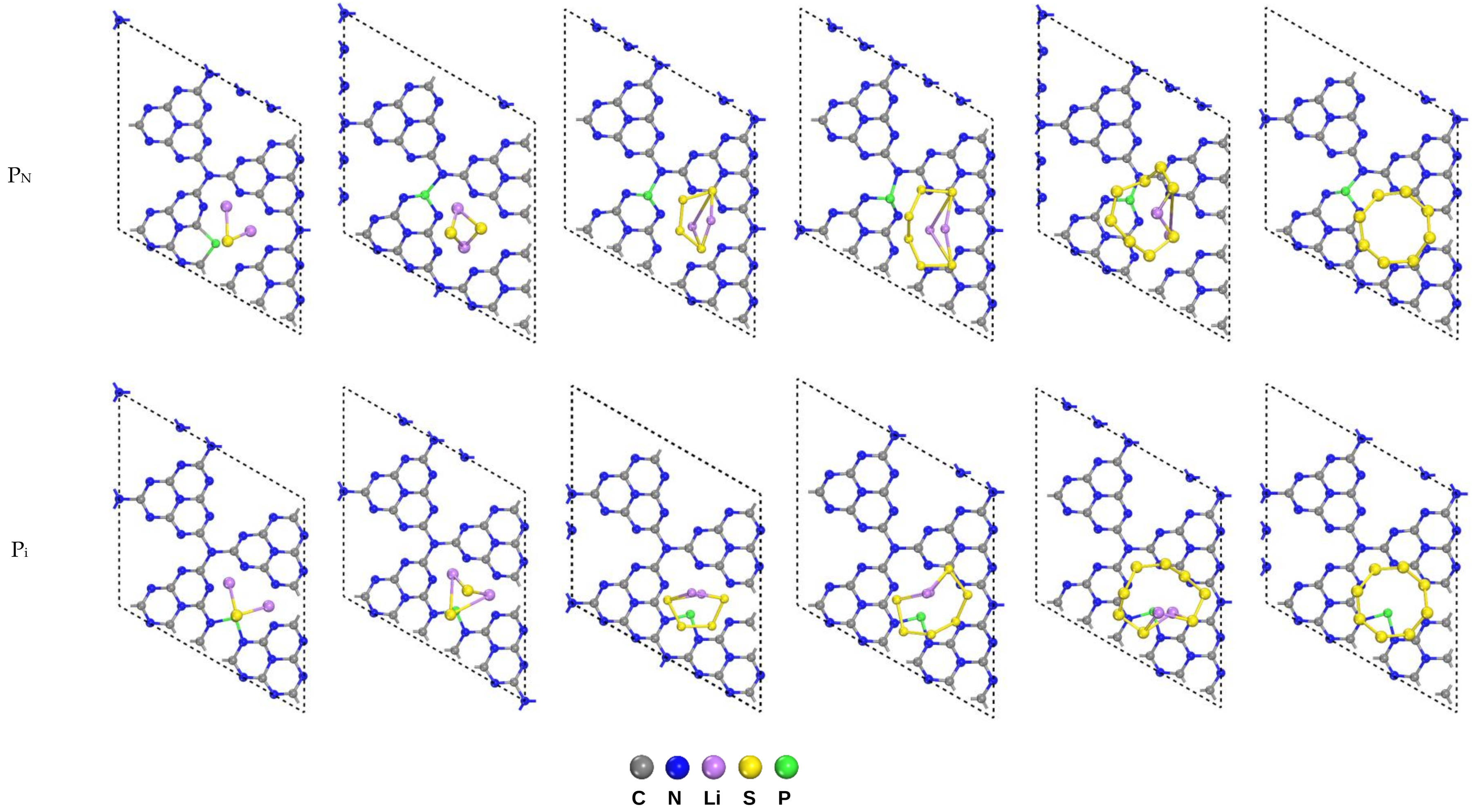
P_N
P_i
C
N
Li
S
P

Table S2. Adsorption energies (E_{ad}) of S_8 and Li_2S_n (n = 1, 2, 4, 6, 8) at lithiation stages on the different substrates

E_{ad}	S_8	Li_2S_8	Li_2S_6	Li_2S_4	Li_2S_2	Li_2S
g-C_3N_4	2.021	3.392	3.198	3.350	3.773	3.809
P_N	2.937	4.128	4.026	4.070	5.179	6.551
P_C	3.502	5.026	5.245	5.225	6.525	6.760
P_i	1.820	2.791	3.045	3.071	4.480	5.753

Table S3. The average distance of LiPSs adsorption on P_N and P_i substrate materials.

Molecules	LiPSs		P_N			P_i		
	S-S	Li-S	Li-S	Li-N	E_{ad}	Li-S	Li-N	E_{ad}
Li_2S	-	2.108	2.427	1.897	6.551	2.413	1.982	5.753
Li_2S_2	2.186	2.240	2.515	1.976	5.179	2.446	1.947	4.480
Li_2S_4	2.093	2.386	2.336	2.250	4.070	2.457	2.251	3.071
Li_2S_6	2.078	2.428	2.411	2.280	4.026	2.503	2.176	3.045
Li_2S_8	2.075	2.419	2.473	2.299	4.128	2.495	2.168	2.791
S_8	2.060	-	-	-	2.937	-	-	1.820

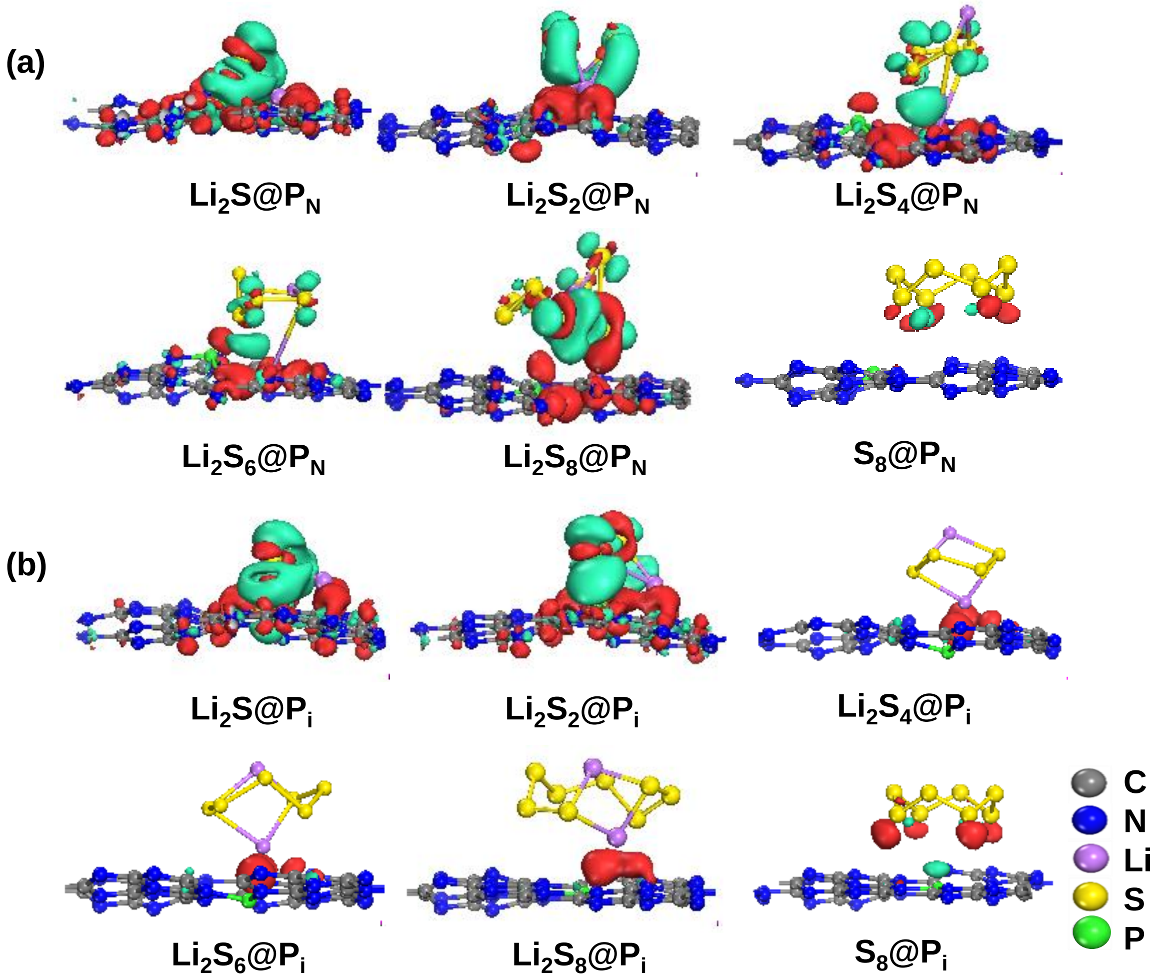

Figure S4. Charge transfers of Li_2S_n (n= 1, 2, 4, 6, and 8) and S_8 clusters adsorbed on P_N (a) and P_i (b) substrates. The green and red iso-surfaces represent electron density loss and gain regions, respectively. The iso-surfaces are 0.004 e/Å^3 for S_8, 0.02 e/Å^3 for Li_2S_n. (n= 1, 2, 4, 6 and 8).

Table S4. The Hirshfield charge analysis of S_8 and Li_2S_n (n = 1, 2, 4, 6, 8) adsorbed on the different substrates with PBE functional.

e	S_8 LiPSs	LiPSs@g-C_3N_4	g-C_3N_4	LiPSs@P_C	P_C	LiPSs@P_N	P_N	LiPSs@P_i	P_i
Li_2S	-0.01	-0.16	-0.15	-0.01	0	0.17	0.18	0.24	0.25
Li_2S_2	-0.02	-0.05	-0.03	0.08	0.10	0.08	0.10	0.12	0.14
Li_2S_4	-0.02	-0.15	-0.13	0.25	0.27	0.25	0.27	-0.22	-0.20
Li_2S_6	-0.03	-0.13	-0.10	0.15	0.18	0.15	0.18	-0.21	-0.18
Li_2S_8	-0.03	-0.23	-0.20	0.13	0.16	0.13	0.16	-0.18	-0.15
S_8	0	0.09	0.09	0.05	0.05	0.05	0.05	0.07	0.07
